# Automated GMP-compatible production of universal CAR Tregs for organ-targeted tolerance induction

**DOI:** 10.1186/s12967-025-07431-0

**Published:** 2025-12-17

**Authors:** Kavitha Lakshmi, Alexandra von Jutrzenka-Trzebiatowski, Liliana Loureiro, Karla Elizabeth González Soto, Katja Peter, José Manuel Marín Morales, Samikshya Santosh Nirmala, Nicole Berndt, Claudia Arndt, Yueyuan Hu, Jing-Wun Li, Claudia Peitzsch, Anna Taubenberger, Rebekka Wehner, Marc Schmitz, Kristina Hölig, Hinrich Abken, Ezio Bonifacio, Martin Bornhäuser, Michael Bachmann, Anja Feldmann, Anke Fuchs

**Affiliations:** 1 Center for Regenerative Therapies Dresden (CRTD), Center for Molecular and Cellular Bioengineering (CMCB), TUD Dresden University of Technology, Dresden, Germany; 2 Mildred Scheel Early Career Center, Faculty of Medicine Carl Gustav Carus, TUD Dresden University of Technology, Dresden, Germany; 3 Helmholtz-Zentrum Dresden-Rossendorf, Institute of Radiopharmaceutical Cancer Research, Dresden, Germany; 4 DKMS Stem Cell Bank, Deutsche Knochenmarkspenderdatei (DKMS), Dresden, Germany; 5 Biotechnology Center, Center for Molecular and Cellular Bioengineering (CMCB), TUD Dresden University of Technology, Dresden, Germany; 6 National Center for Tumor Diseases (NCT), NCT/UCC Dresden, a Partnership Between DKFZ, Faculty of Medicine and University Hospital Carl Gustav Carus, TUD Dresden University of Technology and Helmholtz-Zentrum Dresden-Rossendorf (HZDR), Heidelberg, Germany; 7 Faculty of Medicine Carl Gustav Carus, Institute of Immunology, TUD Dresden University of Technology, Dresden, Germany; 8 German Cancer Consortium (DKTK), Partner Site Dresden and German Cancer Research Center (DKFZ), Heidelberg, Germany; 9 Institute for Transfusion Medicine, German Red Cross Blood Donation Service North-East, Dresden, Germany; 10 Department of Transfusion Medicine, Medical Clinic I (MK1), University Hospital Carl Gustav Carus, Dresden, Germany; 11 Division for Genetic Immunotherapy, Leibniz Institute for Immunotherapy (LIT), Regensburg, Germany; 12 Department of Hematology, Cell Therapy and Medical Oncology at Medical Clinic I (MK1), University Hospital Carl Gustav Carus, Dresden, Germany

**Keywords:** Regulatory T cells, Treg cell therapy, Universal adapter CAR, Precision immunotherapy, Good manufacturing practice, Closed-system manufacturing, Automated expansion

## Abstract

**Background:**

Adoptive transfer of regulatory T cells (Tregs) has demonstrated safety, feasibility and early signs of efficacy in promoting immunological tolerance in inflammatory conditions such as graft-versus-host disease (GvHD). Chimeric antigen receptor (CAR)-engineered Tregs offer localized activation and suppression compared to polyclonal Tregs, but their clinical translation is limited by high manufacturing costs, lengthy developing times and fixed single-antigen specificity. To address these limitations, we employed the universal adapter Reverse CAR (RevCAR) system, which harbors a peptide epitope lacking intrinsic antigen specificity but provides flexibility in targeting through the use of an antigen-specific RevCAR Target Module (RevTM). As a proof-of-concept, we used a RevTM targeting carcinoembryonic antigen (CEA), which is highly expressed in the gastrointestinal (GI) tract, as a potential strategy to achieve localized immunosuppression in GI acute GvHD.

**Methods:**

To support clinical translation, we established an automated, GMP-compatible, clinical-scale manufacturing process. Tregs were magnetically enriched from leukapheresis using the CliniMACS^®^ Plus, followed by high-purity sorting on the MACSQuant^®^ Tyto^®^. The sorted cells were virally transduced and the RevCAR Tregs were expanded on the CliniMACS Prodigy^®^ to obtain clinically relevant cell numbers. The harvested products were evaluated for phenotype, stability, antigen specificity and suppressive function.

**Results:**

Across five manufacturing runs, Tregs (CD4^+^CD25^high^CD127^low^FOXP3^+^) with a median initial purity of 94% were expanded to achieve a median therapeutic yield of 602 × 10^6^ cells. The final product maintained a high purity (median: 91.9%) and exhibited high RevCAR expression (median: 60% RevCAR^+^). Mass cytometry analysis revealed that expanded RevCAR Tregs predominantly exhibited a central memory phenotype with high expression of functional and homing markers. Under experimental pro-inflammatory conditions, the cells maintained stable FOXP3 and Helios expression with minimal pro-inflammatory cytokine production. Importantly, RevCAR Tregs showed antigen-specific activation upon target engagement via the CEA-specific RevTM and robust, dose-dependent suppression.

**Conclusion:**

The study establishes a scalable, GMP-compatible process for manufacturing pure, stable and functional universal RevCAR Tregs for clinical applications. Furthermore, the RevCAR system offers a promising approach toward an allogenic, off-the-shelf Treg therapy capable of treating diverse immune-mediated diseases with spatial precision.

**Supplementary information:**

The online version contains supplementary material available at 10.1186/s12967-025-07431-0.

## Introduction

Autoimmune diseases and transplant-associated immune complications are currently managed primarily with systemic immunosuppressive drugs [1]. However, these drugs typically require long-term administration and their lack of specificity results in the suppression of both harmful and beneficial immune responses. This broad immunosuppressive approach is associated with considerable risks, including an increased susceptibility to infections and cancer, as well as a heightened risk of cardiovascular and renal toxicity, diabetes and obesity [2, 3]. Consequently, there is a crucial need for more targeted immunosuppressive approaches to reduce these risks and improve the quality of life for affected patients.

Over the past two decades, regulatory T cells (Tregs), a subset of T lymphocytes comprising 1–3% of peripheral blood mononuclear cells, have emerged as promising cellular therapies. These ‘living drugs’ are intended to reduce or potentially eliminate the use of traditional immunosuppressive drugs [4]. Clinical studies have demonstrated the safety and feasibility of polyclonal Treg-based therapies in the treatment of graft-versus-host disease (GvHD) [5–10], solid organ transplantation [11–13] and autoimmune diseases such as type 1 diabetes (T1D) [14–16], mostly with early indications of efficacy. However, preclinical studies using animal models suggest that antigen-specific approaches, such as chimeric antigen receptor (CAR)-engineered Tregs, exhibit specific localization and superior potency compared to polyclonal Treg therapies and are therefore currently being explored [17–20].

In an effort to create CAR therapies that can be applied to a broad range of disease indications while saving costs and reducing lengthy development times, universal CAR platforms incorporating adaptor molecules for target recognition have been developed [20–22]. One such approach is the Reverse CAR (RevCAR) system, originally developed for cancer therapy and currently investigated in a clinical phase I trial with patients having CD123^+^ acute myeloid leukemia (NCT05949125) [23–28]. Instead of a conventional single-chain fragment variable (scFv) for antigen recognition, RevCAR T cells are engineered to express a peptide epitope (E5B9 and/or E7B6), which lacks intrinsic antigen specificity and is derived from the nuclear human La/SS-B protein [23, 29, 30]. This alteration reduces the size of the CAR-encoding gene and minimizes risks of non-specific antigen binding and tonic signaling [23]. Antigen specificity is conferred through a bispecific RevCAR Target Module (RevTM), which binds both the peptide epitope on RevCAR T cells and the target antigen. This design enables precise control, as RevCAR T cells can be activated with RevTM administration and deactivated upon its withdrawal, with rapid clearance via kidneys [23, 25].

Given these advantages, the present study set out to generate RevCAR Tregs that could, in principle, be broadly applied to any immune-mediated disease requiring immunosuppression, by administering the appropriate antigen-specific RevTM. As proof-of-concept, we selected acute GvHD (aGvHD) as an ideal disease model for targeted immunosuppression. This choice was based on several factors: (i) lack of efficient and safe immunosuppressive drugs for aGvHD presents a major unmet clinical need [31]; (ii) aGvHD predominantly affects regions of the gastrointestinal (GI) tract, skin and liver, allowing Tregs to be directed to specific inflamed sites [32]; (iii) healthy Tregs with high proliferative capacity can be isolated from the human leukocyte antigen (HLA)-matched hematopoietic stem cell transplantation (HSCT) donors [33]; (iv) prophylactic therapy is feasible in planned HSCT procedures [8, 9] and (v) lymphopenia induced by transplant protocols may enable better Treg expansion, reducing the required therapeutic dose [33]. Among the affected organs, GI aGvHD presents the biggest clinical challenge, as it is one of main causes of morbidity and mortality following allogenic-HSCT [34]. Patients with severe (grade 3–4) GI aGvHD have an overall survival rate of approximately 25% and exhibit a steroid resistance rate as high as 73%, further underscoring the urgent need for alternative therapeutic strategies [35].

In this study, we developed a clinical-scale, good manufacturing practice (GMP)-compatible process for the production of universal RevCAR Tregs that incorporates advanced strategies for Treg isolation and use of a closed, automated system for gene transfer and expansion. As a model for targeted immunosuppression in GI aGvHD, we directed these RevCAR Tregs towards carcinoembryonic antigen (CEA), an antigen highly expressed on the apical surface of GI epithelia, and expressed in a non-polarized fashion under inflammatory conditions [36], thereby becoming visible to tissue-infiltrating Tregs [37, 38]. Moreover, since the RevCAR Treg product itself is universal, the established manufacturing approach holds significant clinical potential not only for aGvHD but also for a multitude of autoimmune and other immune-mediated diseases requiring immune modulation.

## Results

### Design of a RevCAR Treg platform for organ-targeted immunosuppression

**Fig. 1:**
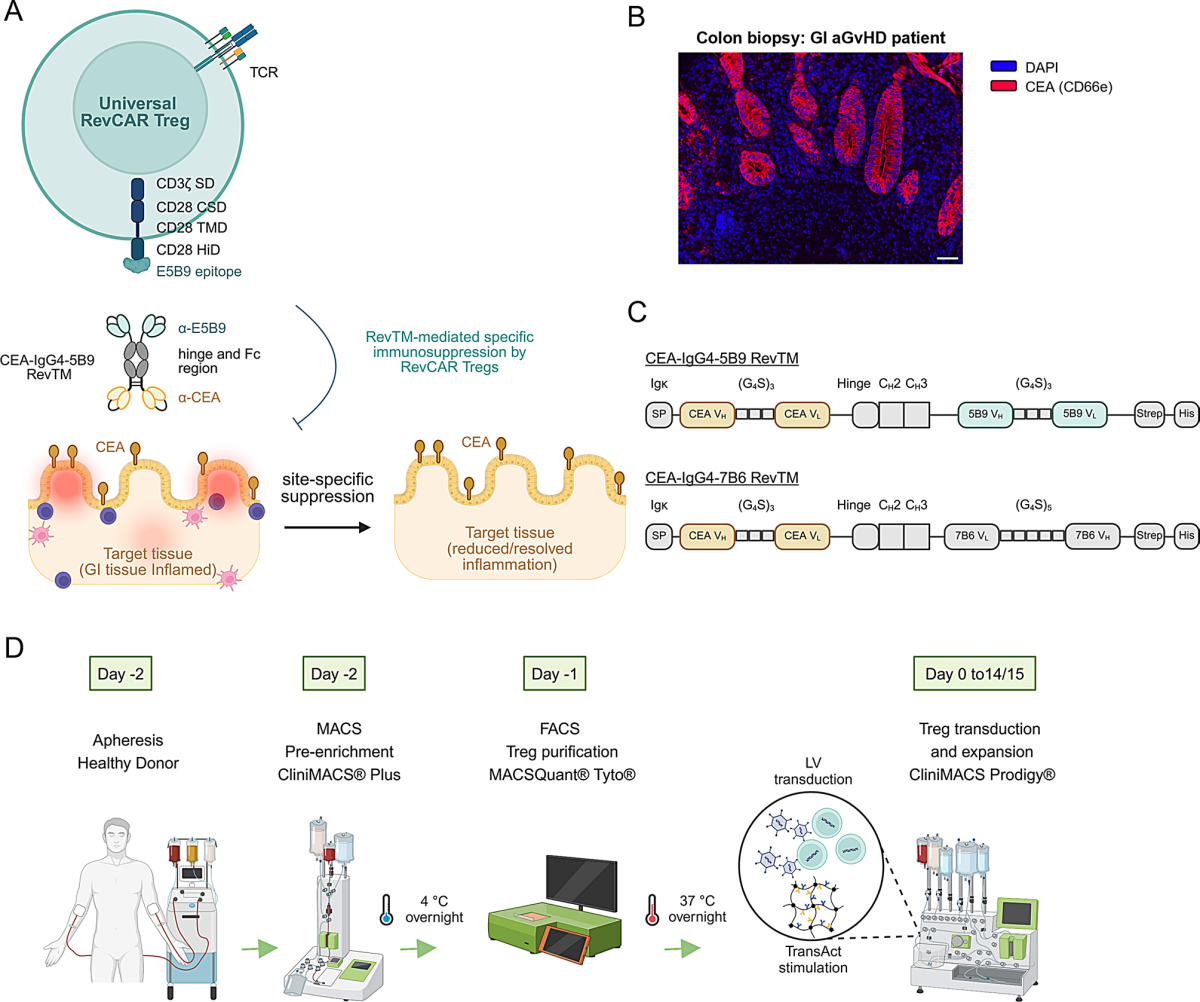
Schematic representation of the RevCAR system and GMP-compatible manufacturing process for RevCAR Tregs. (**A**) RevCAR Tregs harbor a second-generation CAR possessing an extracellular unique peptide epitope (E5B9), hinge (HiD), transmembrane (TMD) and co-stimulatory domain (CSD) of CD28 as well as the signaling domain (SD) of CD3ζ. These Tregs are designed to specifically bind to inflamed GI tissue, a commonly affected organ in aGvHD, through a homodimeric CEA-IgG4-5B9 RevTM that simultaneously binds the E5B9 peptide epitope on RevCAR Tregs and CEA on GI epithelium, facilitating site-specific immunosuppression and reducing tissue inflammation. (**B**) Immunofluorescence staining of a colon biopsy tissue section from a patient with advanced aGvHD (grade 3–4 in the colon and duodenum). Nuclei are stained with DAPI (blue), while CEA is detected using an anti-human CEA antibody followed by detection using Opal620 fluorophore (red). Scale bar: 50 µm. (**C**) Schematic representation of relevant structural components of the CEA-IgG4-5B9 RevTM. The novel CEA-IgG4-5B9 RevTM contains two scFvs with specificity for CEA and E5B9 derived from the variable heavy (V_H_) and light (V_L_) chain of the respective monoclonal antibodies which are connected via glycine (G)-serine (S) linker. Between the two scFvs – the hinge, C_H_2 and C_H_3 regions of a mutated human IgG4 antibody are incorporated. In addition, the CEA-IgG4-5B9 RevTM possesses an Igκ signal peptide (SP) for secretion as well as tags for purification and detection (Strep- and His-tag). Similar scheme is represented for the irrelevant CEA-IgG4-7B6 RevTM. (**D**) Overview of the clinical-scale manufacturing workflow for RevCAR Tregs, employing GMP-compatible instruments and reagents as well as lentiviral (LV) transduction for gene transfer

To develop a versatile Treg product, we employed the universal adapter RevCAR system, which features a second-generation CAR construct incorporating the co-stimulatory domain (CSD) of CD28 and signaling domain (SD) of CD3ζ, along with the unique peptide epitope E5B9 (Fig. 1A). These RevCAR Tregs can be directed to inflamed GI tissues using a CEA-specific RevTM (CEA-IgG4-5B9 RevTM), taking advantage of the increased visibility of CEA in the GI tract under inflammatory conditions [36]. This targeted approach will enable localized RevCAR Treg activation and subsequent immunosuppression, potentially leading to sustained resolution of symptoms (Fig. 1A). Immunofluorescence staining of a colon biopsy tissue section from a patient with advanced GI aGvHD (grade 3–4 in the colon and duodenum) confirmed CEA (CD66e) expression, underscoring its clinical significance as a potential therapeutic target (Fig. 1B).

The novel CEA-specific RevTM for specific targeting contains scFvs specific to CEA [26] and the peptide epitope E5B9 which are connected via a backbone incorporating the hinge and Fc constant regions (C_H_2 and C_H_3) of a mutated human IgG4 antibody [26]. In addition, it possesses an Igk signal peptide for secretion and a Strep- as well as His-tag for purification and detection (Fig. 1C). Due to formation of disulfide bonds between two hinge regions, the novel CEA-IgG4-5B9 RevTM has a homodimer conformation, as schematically depicted in Fig. 1A. In addition, an irrelevant CEA-IgG4-7B6 RevTM [26] that has no specificity to E5B9 RevCAR Tregs was used for this study as a negative control (Fig. 1C).

To comply with European Advanced Therapy Medicinal Products (ATMP) regulatory guidelines, we established a clinical-scale GMP-compatible manufacturing protocol for RevCAR Tregs, integrating an advanced cell-sorting method and a closed automated system for transduction and expansion (Fig. 1D). The process begins with leukapheresis from a healthy donor, followed by a two-step Treg isolation approach (section “Clinical-scale two-step isolation strategy results in a highly pure treg starting product”) comprising magnetic-activated cell sorting (MACS) using the CliniMACS^®^ Plus system (Miltenyi Biotec) and fluorescence-activated cell sorting (FACS) with the MACSQuant^®^ Tyto^®^ Cell Sorter (Miltenyi Biotec). The isolated Tregs are subsequently transduced and expanded using the CliniMACS Prodigy^®^ (Miltenyi Biotec) until clinically relevant cell numbers are achieved (section “Automated clinical-scale RevCAR Treg transduction and expansion ensures high yield and purity with efficient gene transfer”).

### Clinical-scale two-step isolation strategy results in a highly pure Treg starting product

A highly pure Treg starting product is crucial before gene transfer to minimize the risk of arming contaminating effector cells with the CAR. To achieve this, an optimized two-step isolation strategy was developed utilizing the CliniMACS^®^ Plus system for initial CD25 immunomagnetic enrichment, followed by FACS using the fully closed, GMP-compatible MACSQuant^®^ Tyto^®^ Cell Sorter. The following statistics represent data from five independent manufacturing runs, presented as median values with corresponding ranges.

**Fig. 2:**
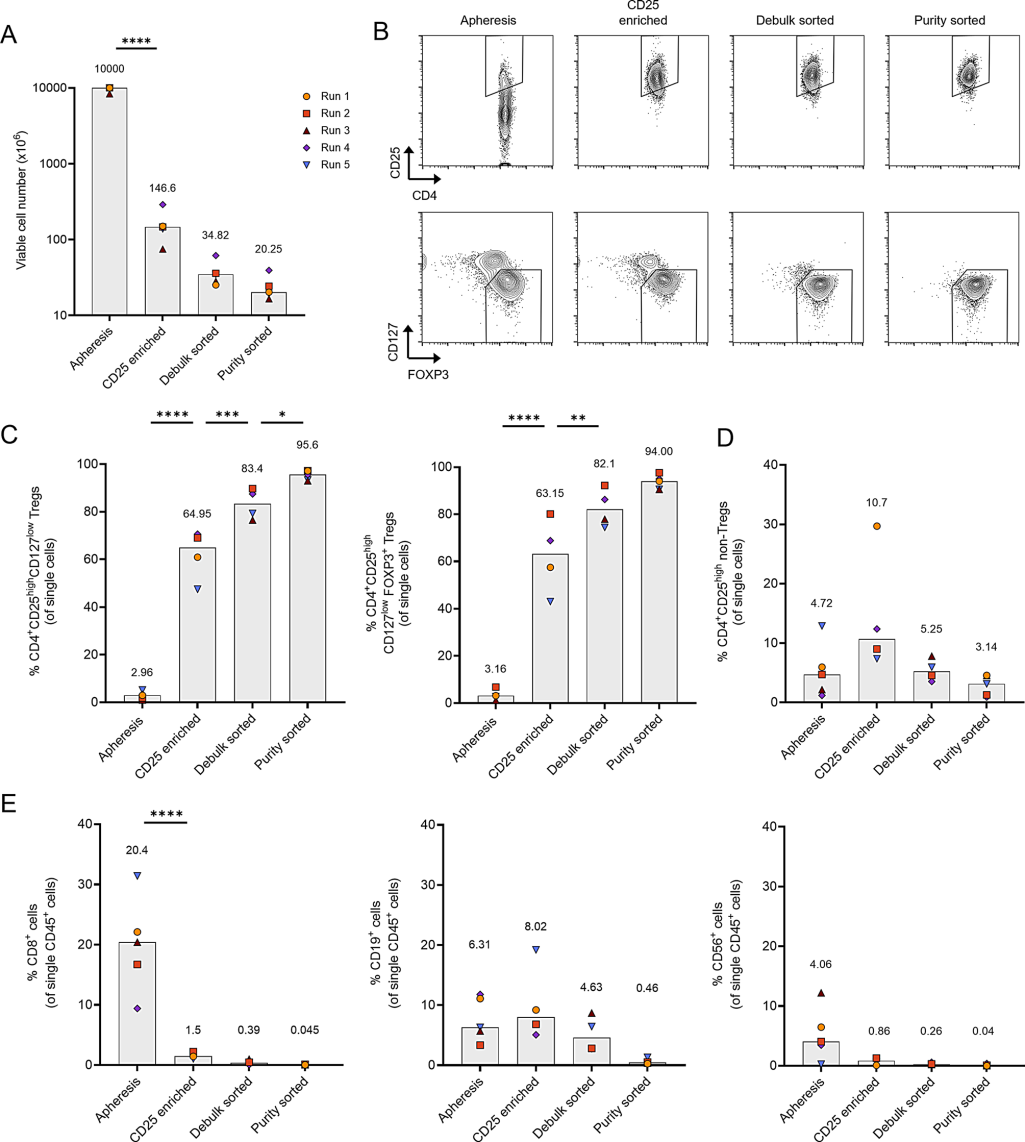
Clinical-scale two-step isolation of Tregs from leukapheresis products. (**A**) Total viable cell numbers selected from the starting apheresis material and the yield obtained after each isolation step, including CD25 enrichment, debulk sorting and purity sorting. (**B**) Representative flow cytometry plots showing CD4^+^CD25^high^ cells (top) and CD127^low^FOXP3^+^ cells gated on CD4^+^CD25^high^ cells (bottom) across the different isolation steps. (**C**) Percentage of CD4^+^CD25^high^CD127^low^ cells (left) and CD4^+^CD25^high^CD127^low^FOXP3^+^ cells (right) within single cells after different isolation steps. (**D**) Percentage of CD4^+^CD25^high^ non-Tregs (defined by CD127^low^FOXP3^+^ NOT gate) within single cells after different isolation steps. (**E**) Percentage of contaminating CD8^+^ T cells (left), CD19^+^ B cells (middle) and CD56^+^ NK cells (right) within CD45^+^ single cells after different isolation steps. Data are derived from five independent runs, with gray bar plots and numbers on top indicating median values. Statistical analysis was performed using repeated measures one-way ANOVA with Tukey’s multiple comparisons test, ****p ≤ 0.0001; ***p ≤ 0.001; **p ≤ 0.01; *p ≤ 0.05

From the obtained apheresis material, 1 × 10^10^ nucleated cells (range: 8.35 × 10^9^ to 1 × 10^10^) were selected for further processing, which resulted in a yield of 146.6 × 10^6^ cells (range: 75 × 10^6^ to 289 × 10^6^) after CD25 immunomagnetic enrichment. Sorting was performed in two stages: (1) two to three ‘debulk’ sorts, for initial enrichment of the target population, and (2) one to two ‘purity’ sorts using lower cell concentrations and stricter gating to maximize product purity (gating strategy in Supplementary Figures S1 and S2). The initial ‘debulk’ sorting resulted in 34.82 × 10^6^ cells (range: 25.23 × 10^6^ to 61.6 × 10^6^) while the ‘purity’ sorting yielded 20.25 × 10^6^ total live cells (range: 16.5 × 10^6^ to 39.4 × 10^6^) (Fig. 2A). This workflow consistently generated a sufficient number of Tregs suitable for expansion.

Flow cytometry analysis confirmed the progressive enrichment of Tregs through each isolation step (Fig. 2B). The frequency of CD4^+^CD25^high^CD127^low^ Tregs increased from 2.96% of total live cells (range: 0.98% to 5.18%) in the apheresis material to 64.95% (range: 47.5% to 70.6%) after CD25 enrichment and further enhanced after FACS sorting − 83.4% (range 76.5% to 89.7%) after debulk sorting and 95.6% (range: 93% to 97.3%) after purity sorting (Fig. 2C, left). Additionally, intracellular FOXP3 staining demonstrated a similar pattern of enrichment, with the proportion of CD4^+^CD25^high^CD127^low^FOXP3^+^ cells starting from 3.16% (range: 1.21% to 6.81%) and enriched to a purity of 94% (range: 90.5% to 97.6%) of total live cells in the final product (Fig. 2C, right).

We also determined the potential contaminating cell populations in these samples (Figs. 2D and 2E). As expected, the proportion of CD4^+^CD25^high^ non-Tregs (identified by a CD127^low^FOXP3^+^ NOT gate), increased from 4.72% to 10.7% of total live cells after CD25 enrichment. However, FACS sorting reduced this contamination to 3.19% (range 0.92% to 4.71%) in the final product. Among CD45^+^ single cells, the frequency of CD8^+^ T cells, initially at 20.4% (range: 9.41% to 31.4%), was reduced to 0.045% (range: 0.011% to 0.12%) in the final sorted product. Similarly, the frequency of CD56^+^ NK cells and CD19^+^ B cells was decreased to 0.04% (range: 0.39% to 0.005%) and 0.46% (range: 1.33% to 0.28%) of total CD45^+^ single cells, respectively. In summary, the implemented GMP-compatible two-step isolation approach reliably yields a highly pure Treg population with minimal contamination, providing an optimal starting material for RevCAR engineering via viral transduction.

### Automated clinical-scale RevCAR Treg transduction and expansion ensures high yield and purity with efficient gene transfer

After isolation, Tregs were expanded and genetically modified using the CliniMACS Prodigy^®^, which ensures sterility and reproducibility with minimal user interaction. Building on our previously established research scale protocols [20] and our first reports of automated polyclonal Treg [39] and RevCAR T cell manufacturing [40] on the CliniMACS Prodigy^®^, we incorporated gene transfer methods during Treg ex vivo expansion to enable RevCAR expression.

**Fig. 3:**
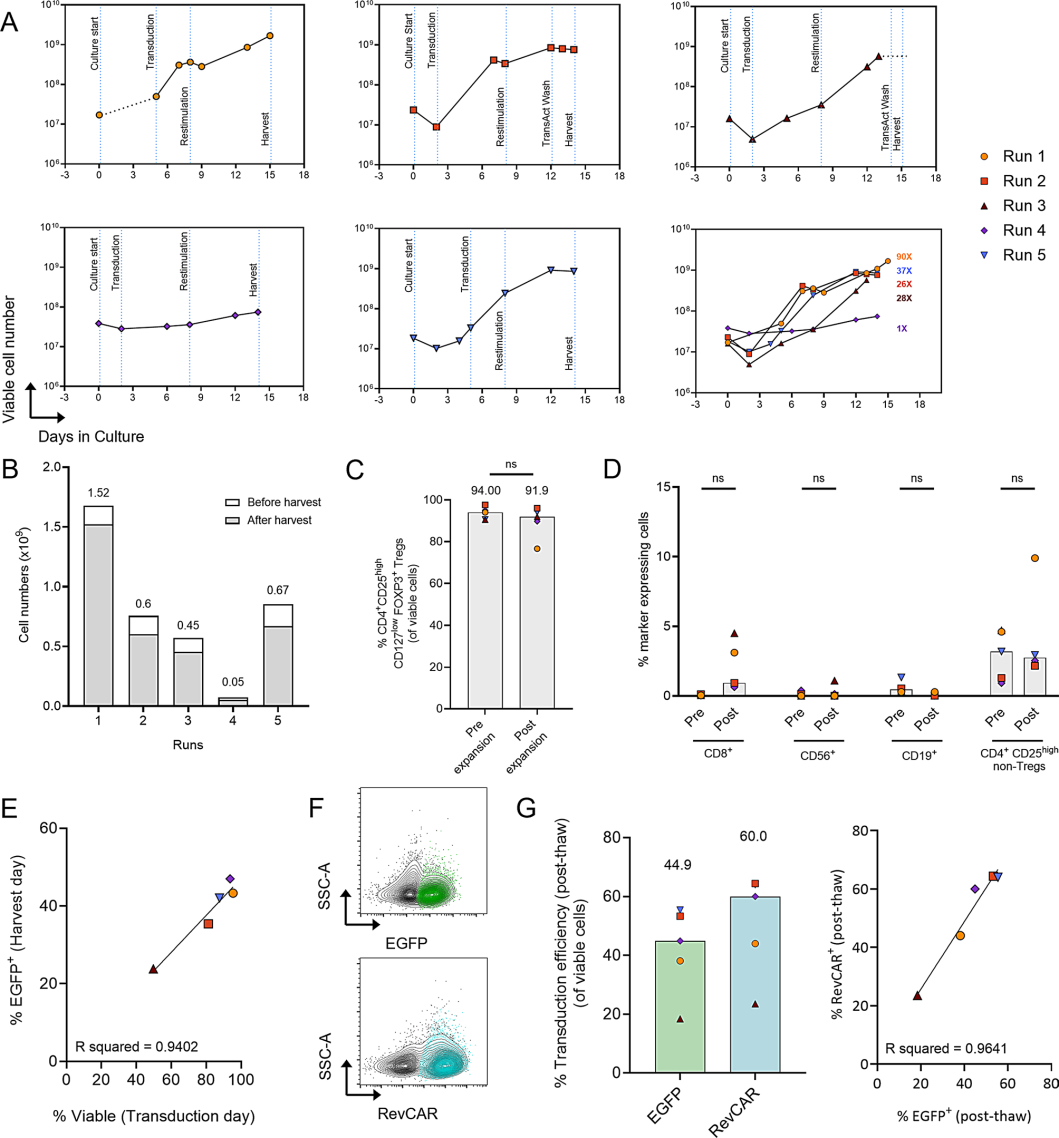
Clinical-scale expansion and transduction of Tregs using the CliniMACS Prodigy^®^ (**A**) Growth curves for five independent runs, with the final plot displaying all runs as overlaid curves and corresponding Fold expansions from Day 0. Vertical dotted lines indicate relevant process steps during expansion. (**B**) Stacked bar plots representing cell yield before harvest (from quality control (QC) pouch, white) and after harvest (from harvest bag, gray). Cell numbers after harvest are indicated on top. (**C**) Percentage of CD4^+^CD25^high^CD127^low^FOXP3^+^ cells within single viable cells at pre-expansion (Day 0) and post-expansion (Day 14/15). (**D**) Percentage of contaminating CD8^+^, CD19^+^ and CD56^+^ within CD45^+^ single viable cells as well as CD4^+^CD25^high^ non-Tregs within single viable cells. (**E**) Correlation between the percentage of viable cells on transduction day and percentage of EGFP^+^ cells on harvest day. (**F**) Representative flow cytometry plots of post-thaw RevCAR treg samples displaying EGFP^+^ cells (green, top) and RevCAR^+^ cells (blue, bottom) overlaid with single viable cells (gray). RevCAR was detected using anti-La monoclonal antibody (5B9). (**G**) Transduction efficiency as determined by the proportion of EGFP^+^ and RevCAR^+^ cells in post-thaw RevCAR Treg products (left), and their correlation (right). Data are derived from five independent runs, with bar plots and values on top indicating median values. Correlation was assessed using Pearson’s correlation coefficient and linear regression analysis. (**C**, **D**) Statistical analysis was performed using a paired t-test; ns, non-significant

Among five independent runs, four exhibited successful expansion with exponential growth, whereas Tregs from Run 4 maintained its cell number without expansion, despite a 14-day culture period, possibly due to donor-related variability (Fig. 3A). Despite this, from all 5 runs, we achieved a median yield of 602 × 10^6^ cells (range: 52.6 × 10^6^ − 1,522.5 × 10^6^) (Figs. 3A and 3B) and a fold expansion of 28x (range: 1x to 90x) (Fig. 3A) after the harvest, sufficient for clinical application. Cell loss during the automated harvest procedure was minimal, with only a slight reduction in cell numbers after harvest (Fig. 3B). The final RevCAR Treg products exhibited viabilities between 73.2% − 97.8% and a high purity, with CD4^+^CD25^high^CD127^low^FOXP3^+^ cells comprising 91.9% (range: 76.6% − 96%). Furthermore, the contamination from undesired cell populations remained minimal (Figs. 3C and 3D). Among CD45^+^ single live cells, the proportion of CD8^+^ T cells was 0.9% (range: 0.62% − 4.5%) and similarly proportions of CD56^+^ NK cells and CD19^+^ B cells were at 0.004% and 0.018%, respectively. CD4^+^CD25^high^ non-Treg cells also remained low at 2.74% of total live cells (range: 2.20% − 10.20%).

Tregs were successfully genetically modified, achieving a high transduction efficiency of 42.1% (range: 23.8% to 47.0%), as assessed by EGFP expression. Notably, the efficiency of genetic modification was strongly influenced by cell viability on the day of transduction (R^2^ = 0.94) (Fig. 3E). Thus, to avoid periods of decreased viability observed during the lag phase of the growth curve (Fig. 3A), we modified the protocol for Run 4 and 5 by allowing flexibility in the timing of transduction. In these cases, lentiviral particles were added only when viability exceeded 75% (Run 4: Day 2, Run 5: Day 5). Furthermore, analysis of freeze-thawed samples confirmed high RevCAR expression (median: 60%) which strongly correlated with the frequency of EGFP^+^ cells (median: 44.9%, R^2^ = 0.96) (Figs. 3F and 3G). Thus, the expansion process resulted in a clinically relevant yield with high Treg purity and efficient gene modification, resulting in robust RevCAR expression.

### Expanded RevCAR Tregs exhibit markers linked to suppressive function, central memory identity and tissue homing

**Fig. 4:**
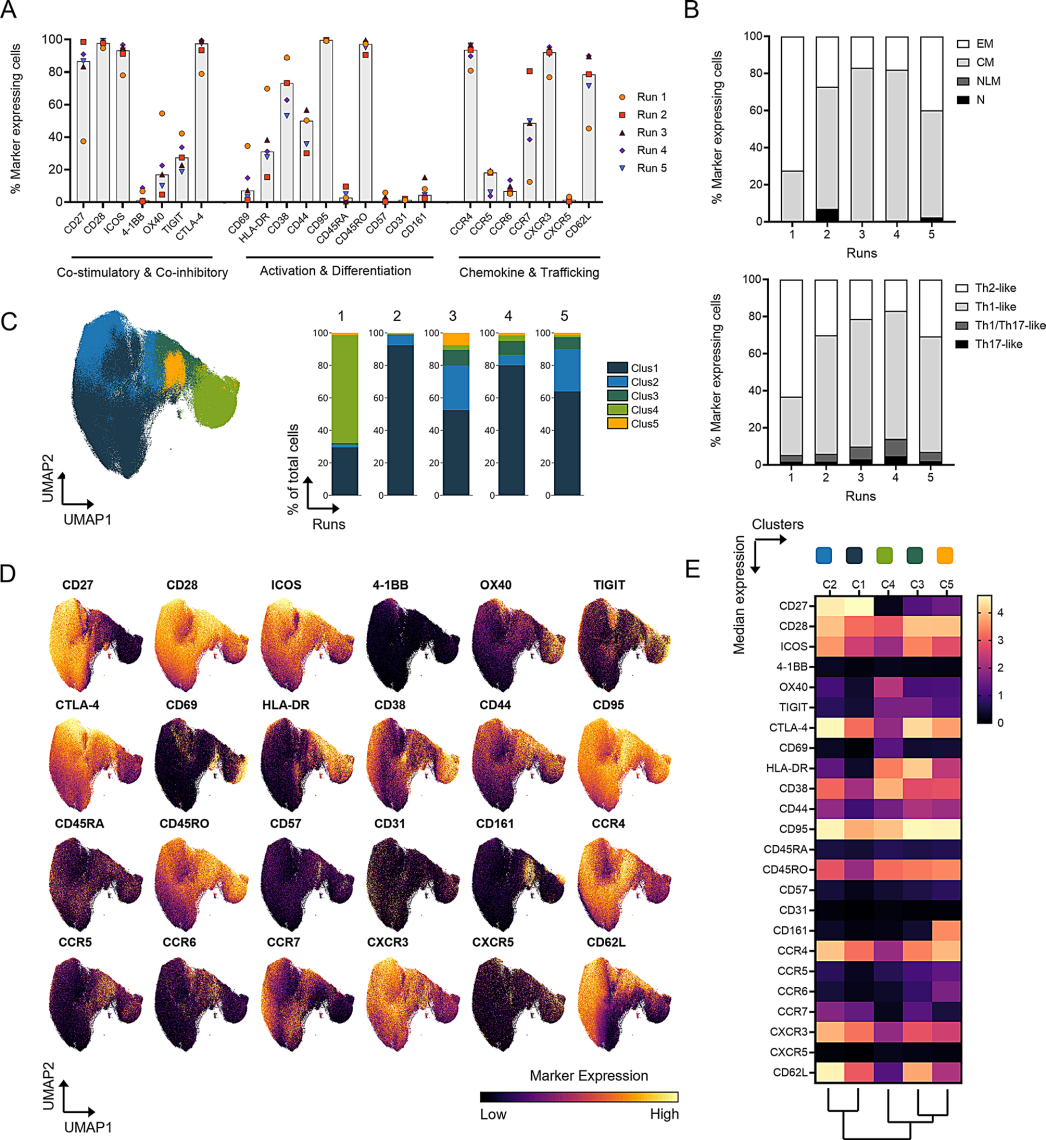
Extended phenotyping of RevCAR Tregs using a 29-marker mass cytometry panel. All analyses were performed on gated CD4^+^CD25^high^CD127^low^FOXP3^+^ Tregs, identified using a standard mass cytometry gating strategy (see Supplementary Figure S3). (**A**) Percentage of cells expressing indicated Treg-relevant marker within the Treg population across the five runs. Bar plots indicate median values. (**B**) Proportion of memory subsets (top): effector memory (EM: CD45RO^+^ CD95^+^ CD62L^−^), central memory (CM: CD45RO^+^ CD95^+^ CD62L^+^), naïve-like memory (NLM: CD45RA^+^ CD95^+^ CD62L^+^) and naïve (N: CD45RA^+^ CD95^−^ CD62L^+^) characterized according to Wendering et al. [46] and Filoni et al. [47]. The proportion of Th-like subsets (below): Th1-like (CXCR3^+^ CCR6^−^), Th2-like (CXCR3^−^ CCR6^−^), Th17-like (CXCR3^−^ CCR6^+^) and Th1/Th17-like (CXCR3^+^ CCR6^+^). (**C**) UMAP representation of all five runs, overlaid and colored according to the five clusters (Clus 1–5) identified through FlowSOM unsupervised clustering using 29 markers. Adjacent stacked bar plots represent the proportion of FlowSOM-based clusters in each Treg product. (**D**) UMAP representation of all five runs, overlaid and colored according to intensity of Treg-relevant marker expression. (**E**) Heatmap summarizing the median expression of the same markers across the five identified clusters, accompanied by a dendrogram reflecting hierarchical clustering relationships

For a comprehensive phenotypic characterization of expanded RevCAR Treg products, a 29-marker mass cytometry panel was utilized. CD4^+^CD25^high^CD127^low^FOXP3^+^ Tregs from Runs 2–5 showed a high frequency ( > 80%) of cells expressing the co-stimulatory molecules CD27 and CD28, as well as the co-inhibitory molecules ICOS and CTLA-4, which are important for Treg proliferation and function [41]. In contrast, Run 1 showed a lower frequency of cells expressing these functional markers (Fig. 4A). Analysis of activation and differentiation markers revealed variable activation and memory states among the RevCAR Tregs. CD69 was expressed on fewer than 15% of cells in Run 2–5, but nearly 35% of cells in Run 1. HLA-DR and CD38 were present on 15–40% and 50–90% of cells respectively, in Runs 2–5, with higher frequencies in Run 1. These indicate a more activated phenotype in Run 1. CD44, linked to Treg differentiation and suppression [42], was expressed on 30 to 60% of cells across all runs. As expected for expanded peripheral blood Tregs, a very high proportion of cells ( > 95%) expressed CD95 and CD45RO and lacked CD31 expression [11, 43]. Notably, the absence of CD57 expressing cells in all products suggested that the RevCAR Tregs were not terminally differentiated or exhausted. Chemokine receptor expressions suggest strong homing potential, with a high frequency of cells ( > 80% of cells, except Run 1) expressing CCR4 (associated with skin homing) and CXCR3 (associated with homing to inflamed tissues) [44, 45]. CCR7 and CD62L were shown by moderate to high frequency of cells (50–90%, except Run 1), suggesting a capacity for lymph node homing.

Furthermore, RevCAR Treg were characterized according to Wendering et al. [46] and Filoni et al. [47] by their memory phenotype: effector memory (EM: CD45RO^+^ CD95^+^ CD62L^−^), central memory (CM: CD45RO^+^ CD95^+^ CD62L^+^), naïve-like memory (NLM: CD45RA^+^ CD95^+^ CD62L^+^) and naïve (N: CD45RA^+^ CD95^−^ CD62L^+^). The products mainly demonstrated a CM phenotype, except for Run 1, which had a higher proportion of EM cells (Fig. 4B, top). In addition, the T helper (Th)-like functional phenotype was assessed: Th1-like (CXCR3^+^ CCR6^−^), Th2-like (CXCR3^−^ CCR6^−^), Th17-like (CXCR3^−^ CCR6^+^) and Th1/Th17-like (CXCR3^+^ CCR6^+^) (Fig. 4B). The RevCAR Tregs predominantly exhibited a Th1-like phenotype (Runs 2–5), whereas Run 1 was enriched for Th2-like cells (Fig. 4B, bottom).

Unsupervised clustering using FlowSOM for 29 markers, along with UMAP visualization, identified five distinct clusters (Clus 1–5) on the gated RevCAR Tregs (Figs. 4C–4E). Clus 1 and 2, predominant clusters in Runs 2–5 (together ≥80%), exhibited strong functional and homing characteristics, with high expression of CD27, CD28, ICOS, CTLA-4, CCR4, CXCR3 and CD62L. In contrast, Run 1 was primarily composed of Clus 4 ( > 60%), which exhibited elevated activation marker expression (CD69, HLA-DR and CD38) and reduced levels of CD27, ICOS, CTLA-4, CCR4, CXCR3, CCR7 and CD62L. Clus 3 and 5 likely represent intermediate or transitionary phenotypes between Clus 1–2 and Clus 4. Importantly, Clus 5, characterized by CD161 expression and associated with a pro-inflammatory phenotype, constituted only a minor proportion of the total cell population in all runs. Overall, RevCAR Treg products (specially from Runs 2–5) display a phenotype favorable for clinical application, with high expression of markers associated with suppressive function, central memory identity and tissue-homing potential.

### RevCAR Tregs are specifically activated upon targeted stimulation

It is crucial that RevCAR Tregs are selectively activated at the target site upon RevTM-mediated recognition of antigen to minimize the risk of off-target immunosuppression. To evaluate this, RevCAR Tregs and the novel CEA-IgG4-5B9 RevTM were cultured in the presence or absence of HT-29 CEA^high^ target cells. Additional controls included an irrelevant CEA-IgG4-7B6 RevTM as well as a condition without RevTM.

**Fig. 5:**
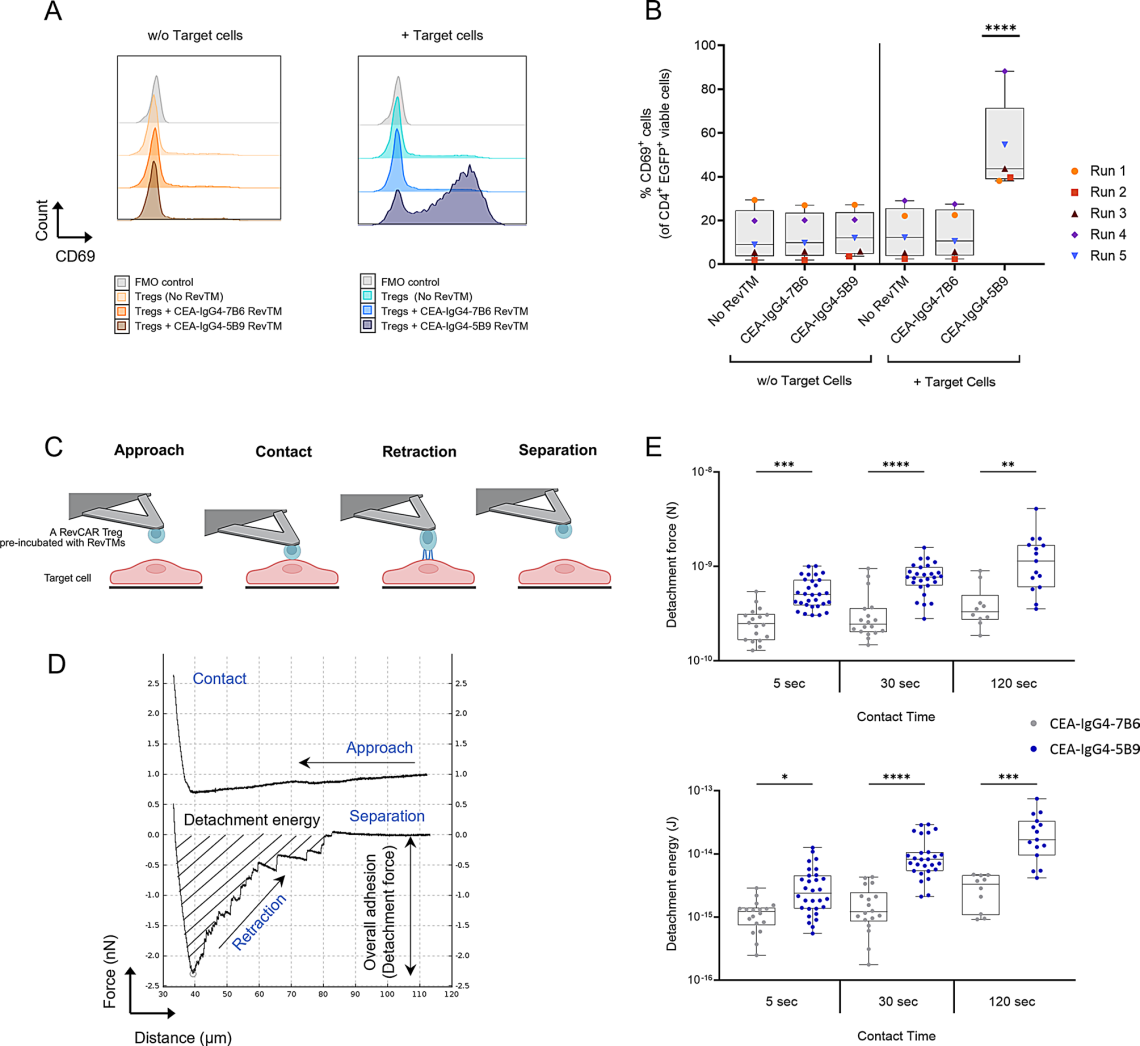
Antigen-specific activation and adhesion of RevCAR Tregs in the presence of target cells and CEA-IgG4-5B9 RevTM. To assess antigen-specific activation, RevCAR Tregs were cultured with CEA-IgG4-5B9 RevTM in the presence or absence of HT-29 CEA^high^ target cells. Control conditions include the irrelevant CEA-IgG4-7B6 RevTM and no RevTM. (**A**) Representative histograms showing CD69 expression in comparison to the fluorescence-minus-one (FMO) control (gated on CD4^+^ EGFP^+^ viable cells). Histograms are normalized to mode. (**B**) %CD69^+^ expressing cells among CD4^+^ EGFP^+^ viable population. Significance represented in comparison to all other conditions (**C**) Schematic representation of atomic force microscopy (AFM)-based single-cell force spectroscopy (SCFS). (**D**) A representative force-distance curve generated from SCFS, illustrating approach, contact, retraction and separation phases. (**E**) Detachment force (top) and detachment energy (bottom) for a single sorted EGFP^high^ RevCAR treg product (Run 1) tested in the presence of CEA-IgG4-5B9 RevTM or CEA-IgG4-7B6 RevTM across different contact durations (5s, 30s, 120s). A total of six RevCAR Tregs (three for specific and three for irrelevant condition) were picked and each cell was probed against three different HT-29 CEA^high^ target cells. Measurements were performed in duplicate for 120s duration and in triplicate for 5s and 30s durations. Statistical analysis was performed by using (**B**) repeated measures two-way ANOVA with Tukey’s multiple comparisons test or (**E**) Kruskal-Wallis test with Dunn’s correction for multiple comparisons, ****p ≤ 0.0001; ***p ≤ 0.001; **p ≤ 0.01; *p ≤ 0.05

After 24 hours of culture, RevCAR Treg cells were analyzed for CD69 expression, an early activation marker. Results demonstrate that RevCAR Tregs were specifically activated in the presence of CEA-IgG4-5B9 RevTM and HT-29 CEA^high^ target cells, with a median of 43.67% CD69^+^ expressing cells within the CD4^+^ EGFP^+^ viable population (Figs. 5A and 5B). In contrast, no increase in the frequency of CD69^+^ expressing cells was observed in the presence of the irrelevant RevTM.

Adhesion forces within a CAR-antigen complex play a crucial role in mechanotransduction and downstream signaling cascades [48]. A stronger adhesion force between RevCAR Tregs and HT-29 CEA^high^ target cells in the presence of the CEA-IgG4-5B9 RevTM in comparison to irrelevant RevTM would therefore provide further evidence for specific binding and activation. Furthermore, increased adhesion forces would indicate that these RevCAR Tregs can efficiently recognize the target antigen and be retained at the inflammation site.

To investigate this, we employed atomic force microscopy (AFM)-based single-cell force spectroscopy (SCFS) to quantify and compare adhesion forces between individual RevCAR Tregs and target cells in the presence of either the specific CEA-IgG4-5B9 RevTM or the irrelevant CEA-IgG4-7B6 RevTM. To ensure that only transduced cells were selected, the RevCAR Tregs were sorted based on CD4^+^CD25^high^CD127^low^EGFP^high^ expression. A concanavalin A (Con A)-coated cantilever was used to pick up a single EGFP^high^ RevCAR Treg pre-incubated with either of the two RevTMs. The Treg cell was then positioned above an HT-29 CEA^high^ target cell and gradually lowered to establish contact. Cells remained in contact for defined durations (5s, 30s and 120s) before the cantilever was retracted until complete separation (Fig. 5C). Force-distance curves generated from these measurements allowed us to calculate detachment force (N) and detachment energy (J) under both conditions (Fig. 5D). As expected, irrespective of the RevTM used, EGFP^high^ RevCAR Tregs exhibited increasing detachment force and detachment energy over time, indicating progressive stabilization of cell-cell interactions. Importantly, however, for all probed contact times, the adhesion was stronger in the presence of specific CEA-IgG4-5B9 RevTM compared to irrelevant CEA-IgG4-7B6 RevTM (detachment force: a median increase from 0.50 to 1.14 nN with CEA-IgG4-5B9 RevTM versus 0.25 to 0.33 nN with CEA-IgG4-7B6 RevTM, detachment energy: a median increase from 2.4 × 10^15^ J to 16.9 × 10^15^ J with CEA-IgG4-5B9 RevTM versus 1.2 × 10^15^ J to 3.3 × 10^15^ J with CEA-IgG4-7B6 RevTM) (Fig. 5E).

### RevCAR Tregs maintain a stable phenotype under pro-inflammatory conditions

**Fig. 6:**
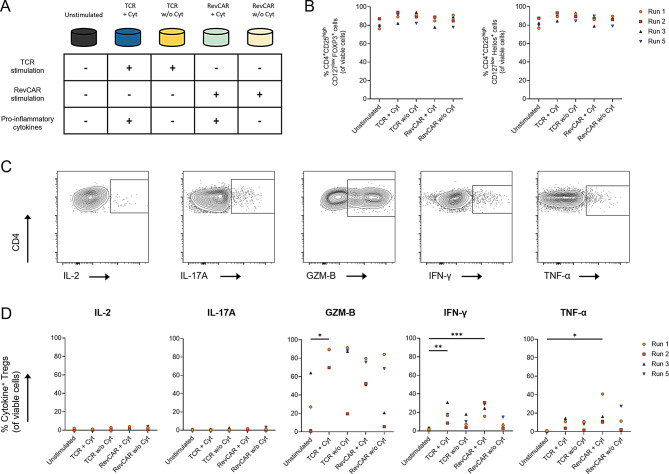
Characterization of phenotypic stability of RevCAR Tregs under pro-inflammatory conditions. RevCAR Tregs were cultured for 48 hours under different stimulatory conditions with (+ Cyt) and without (w/o Cyt) addition of a cytokine cocktail composed of IL-1β, IL-12, IL-23, IL-6 and TNF-α and analyzed using flow cytometry. TCR stimulation was induced using Dynabeads™ human T-Activator CD3/CD28, whereas RevCAR stimulation was performed by co-culturing the RevCAR Tregs with CEA-IgG4-5B9 RevTM and HT-29 CEA^high^ target cells. (**A**) Schematic representation of experimental conditions. (**B**) Percentage of CD4^+^CD25^high^CD127^low^FOXP3^+^ Tregs (left) and CD4^+^CD25^high^CD127^low^Helios^+^ Tregs (right) within single viable cells across the five conditions. (**C**) Representative flow cytometry plots illustrating the gating strategy for each corresponding cytokine after pre-gating on CD4^+^CD25^+^CD127^low^ Tregs. (**D**) Intracellular cytokine staining showing the percentage of cytokine-producing cells within CD4^+^CD25^+^CD127^low^ single viable cells. (**B**, **D**) Statistical analysis was performed using repeated measures one-way ANOVA with Tukey’s multiple comparisons test, ***p ≤ 0.001; **p ≤ 0.01; *p ≤ 0.05

Given that RevCAR Tregs would localize at the target sites with high inflammation in vivo, it is imperative to evaluate their phenotypic stability under pro-inflammatory conditions. For this purpose, expanded RevCAR Tregs from the four successful expansions (Runs 1, 2, 3 and 5) were cultured under different stimulation conditions and a pro-inflammatory cytokine cocktail composed of IL-1β, IL-12, IL-23, IL-6 and TNF-α (Fig. 6A). Flow-cytometry read-out showed that expanded RevCAR Tregs maintained high FOXP3 and Helios expression levels across different conditions (Fig. 6B). In addition, intracellular cytokine analysis showed no IL-2 or IL-17A production and only a slightly elevated secretion of IFN-γ and TNF-α under pro-inflammatory conditions (Figs. 6C and 6D). Notably, expanded but unstimulated RevCAR Tregs exhibited low to intermediate levels of granzyme B expression, which in some cases increased upon stimulation – a pattern previously reported [49, 50] – and aligning with its established role in Treg-mediated suppression.

In summary, RevCAR Tregs show a stable favorable phenotype for a clinical application, as they are able to retain the FOXP3 and Helios expression with minimum secretion of pro-inflammatory cytokines and pronounced secretion of granzyme B, even under proinflammatory conditions.

### RevCAR Tregs maintain suppressive function after RevCAR engineering and expansion

**Fig. 7:**
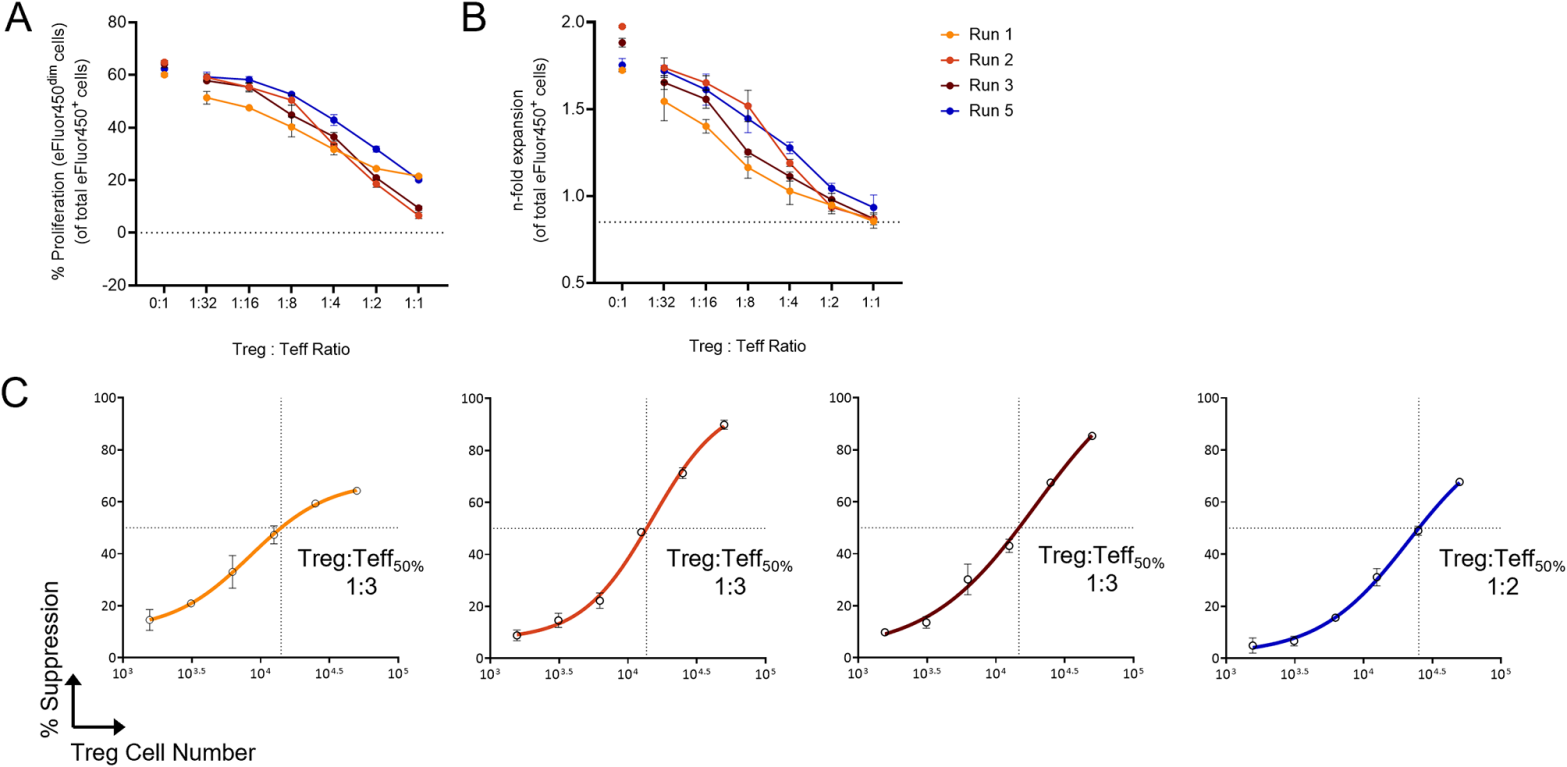
In vitro suppressive capacity of RevCAR Tregs. eFluor670-stained RevCAR Tregs from four successful expansions were co-cultured with eFluor450-stained CD4^+^CD25^-^ T effector cells (Teffs) isolated from a single allogeneic donor at the indicated ratios under CD3/CD28 polyclonal stimulation for four days. As controls, eFluor450^+^ Teffs were incubated alone in the presence (0:1 Treg:Teff ratio) or absence (dotted line in (**A**) and (**B**) representing mean of unstimulated Teffs) of a CD3/CD28 polyclonal stimulation. (**A**) Percentage proliferation was assessed as the proportion of eFluor450^dim^ cells within the total eFluor450^+^ Teffs. (**B**) n-fold expansion was calculated using cell counts of eFluor450^+^ Teffs on Day 4 relative to Day 0. (**C**) Percentage suppression was determined as described in the methods section. A nonlinear four-parameter dose-response curve was applied to calculate Treg numbers required for 50% suppression of Teff proliferation (Teff_50%_) for each run and corresponding Treg:Teff_50%_ ratios are presented

A crucial step in evaluating the RevCAR Tregs was to confirm their suppressive functionality following genetic engineering and expansion. To enable a direct comparison across all runs, eFluor670-stained RevCAR Tregs from the four successful expansions (Runs 1, 2, 3 and 5) were co-cultured with eFluor450-stained CD4^+^CD25^-^ T effector cells (Teffs) as responder cells from a single allogeneic donor under CD3/CD28 polyclonal stimulation. After four days, flow cytometry analysis demonstrated that all expanded RevCAR Tregs exerted potent, dose-dependent suppression of Teff proliferation, as measured by eFluor450 dilution (Fig. 7A), and overall Teff expansion, based on total eFluor450^+^ cell counts (Fig. 7B). To quantify and compare the suppressive capacity of the RevCAR Treg products, Treg cells required for 50% suppression of Teff proliferation (Teff_50%_) were calculated and corresponding Treg:Teff_50%_ ratios were determined (Fig. 7C). Run 1, 2 and 3 had comparable Treg:Teff_50%_ ratio of 1:3, whereas Run 5 required slightly higher doses, with Teff_50%_ occurring at 1:2 ratio. To additionally assess whether gene modification influenced suppressive function, we compared the suppressive activity of transduced and non-transduced cells within the same product. For this purpose, RevCAR Tregs from Run 2 were sorted based on CD4^+^CD25^high^ expression and the transduction marker EGFP. Subsequently, a suppression assay using CD3/CD28 polyclonal stimulation was performed with EGFP^+^ and EGFP^-^ sorted Tregs, co-cultured with autologous Teffs for four days. The results demonstrate robust suppressive activity between both transduced and non-transduced Tregs, indicating that genetic modification did not impair their regulatory function (Supplementary Figure S4).

## Discussion

CAR Tregs hold significant potential in adoptive immunotherapy, offering targeted localization, superior suppressive capacity and enhanced ability to prevent graft rejection and GvHD development compared to polyclonal Tregs [18, 51, 52]. Despite this, CAR Tregs with a single specificity have limited therapeutic applicability and are costly and time-consuming to develop [53, 54]. A promising strategy to overcome these challenges is utilizing our previously developed universal adapter RevCAR platform, which allows the rapid adjustment of antigen specificity through the exchange of RevTM. In addition, the universal nature of this platform enables the development of off-the-shelf Treg products – either by selecting HLA-homozygous donors, making them compatible with a large proportion of patients across multiple disease indications, or through further genetic engineering [55]. As an initial proof-of-concept, the system was designed for the treatment of GI aGvHD, a condition that remains particularly difficult to manage and is implicated in nearly all fatal cases of aGvHD [34, 35]. The target antigen used in this study, CEA, is highly expressed in the GI tract, predominantly on the luminal site. However, under inflammatory conditions, its expression becomes non-polarized, making it accessible to immune cells [36, 37, 56]. We hypothesize that this would result in localized immunosuppression upon adoptive transfer of RevCAR Tregs with CEA-specific RevTMs.

To support future clinical application, we established a GMP-compatible, clinical-scale protocol to manufacture universal RevCAR Tregs. To achieve this, we had to address the initial purity of Treg after isolation. Existing clinical-scale Treg isolation protocols that rely on CD25 enrichment with additional depletion of CD8^+^ and optionally CD19^+^ cells, typically report purities between 60 and 90% [6, 56–59]. While this level of purity is acceptable for polyclonal Treg applications and has not been linked to adverse effects, achieving higher purity was critical in our study to prevent the unintended arming of contaminating conventional T cells with a CAR, which could augment the inflammation and amplify the disease state at the target site. However, conventional FACS sorting to enhance purity presents challenges, including difficulties meeting ATMP guidelines in many countries, lengthy processing times and extensive operator involvement [60–62]. To address these issues, we utilized the closed-system MACSQuant^®^ Tyto^®^ Cell Sorter with high-speed cartridges for automated, operator-free sorting, following CD25 pre-enrichment from apheresis material. Using this approach, we successfully sorted a median value of 146.6 × 10^6^ cells in only 2.3 hours (average) through two ‘debulk’ sorts, followed by an additional ‘purity’ sort lasting 1.25 hours (average), with minimal operator intervention. This method consistently achieved a high Treg purity (median 94%), as assessed by FOXP3 expression.

Building upon our previously established protocols for expansion of polyclonal Tregs and RevCAR T cells on the CliniMACS Prodigy^®^ [39, 63], as well as a proof-of-concept research-scale study for UniCAR Tregs [20], we further optimized the process to enable large-scale Treg manufacturing while incorporating gene transfer and a soluble stimulation reagent, T Cell TransAct^™^. Using this refined protocol, we could reach therapeutically relevant numbers with a high purity (median 91.9% of CD4^+^CD25^high^CD127^low^FOXP3^+^ cells), allowing for the administration of a median target dose of 8.6 × 10^6^ cells/kg body weight for a standard 70 kg patient. Notably, even in Run 4, where RevCAR Tregs failed to expand, the process still yielded 52.6 × 10^6^ cells with high purity (89.7% CD4^+^CD25^high^CD127^low^FOXP3^+^ cells) and a transduction efficiency of 60% (RevCAR^+^), with a permissible dose of 0.75 × 10^6^ cells/kg for a standard 70 kg patient. Previous studies evaluating polyclonal Tregs have demonstrated that doses ranging from 0.5–10 × 10^6^/kg, and in some cases up to 30 × 10^6^/kg, have been well tolerated in clinical settings [64]. While preclinical data suggest that antigen specificity of CAR Tregs may enable lower dosing [65], excessively low doses could also likely result in reduced persistence and efficacy [66, 67]. Ongoing clinical trials, such as the dose-escalation study in kidney transplant patients receiving HLA-A2 CAR Tregs ranging from 10^4^ to 10^9^ per kg body weight (NCT04817774), are expected to provide better insights into the optimal therapeutic CAR Treg dose [68].

Comprehensive phenotypic characterization using mass cytometry showed that expanded RevCAR Tregs expressed high levels of co-stimulatory and co-inhibitory receptors such as CD27, CD28, ICOS and CTLA-4. These molecules are critical for Treg functionality and stability: CD27 has been associated with enhanced suppressive potency and inhibition of Treg-to-Th17 conversion [69, 70] while CD28, ICOS and CTLA-4 are well-established markers of Treg maintenance and suppressive functionality [71–73]. Additionally, the high expression of chemokine receptors such as CCR4 and CXCR3 suggests strong homing potential to skin [74] and inflamed tissues [75, 76]—which are specially relevant to GvHD pathology. In particular, CD62L expression indicates the capacity for lymph node trafficking which has been shown to enhance bone marrow engraftment and protection from GvHD in preclinical models [77, 78]. Furthermore, the predominant CM phenotype seen in most runs has been associated with superior suppressive function and enhanced in vivo persistence due to its high proliferative capacity [79]. The high frequency of Th1-like RevCAR Tregs suggests that they may preferentially suppress Th1 effector responses, such as IFN-γ–mediated inflammation, while in mouse models, these cells have also been associated with protection against allograft rejection [80].

Run 1 deviated from the aforementioned phenotype observed in other runs, with lower expression of several key functional and homing markers, increased EM phenotype and a higher representation of Clus 4 in the unsupervised clustering analysis—which was also characterized by elevated activation and differentiation markers. This distinct phenotype in Run 1 is likely a result of three main reasons: (1) restimulation (Day 8) with a higher concentration of TransAct (130%), likely causing excessive expansion, which has previously been linked to loss of CD27 (as also seen by us) and gain of CD70, correlating with reduced suppressive function [81]; (2) high cell concentration during culture period due to fixed maximal culture volume (8.6 × 10^6^ cells/mL in Run 1 vs. 2.65 × 10^6^ cells/mL median in other runs). This has also been shown by MacDonald et al. (2022) to impair FOXP3 expression and viability, effects that could be mitigated by refreshing culture medium [82]. However, to avoid complete medium change, we suggest a partial exchange at short intervals towards the end of culture to ensure sufficient nutrient availability. And finally, (3) lack of an additional wash step to remove residual stimulatory agents before final harvest. In line with our findings, recent clinical data from Bender et al. (2024) also suggest that limiting fold expansion may improve therapeutic potency, as lower-expanded Treg products in their phase II T1D clinical trial showed better C-peptide preservation, likely due to increased in vivo persistence [16]. Similarly, another study highlighted that strong expansion can also alter the methylation status of Tregs, which can lead to Treg instability [83]. In summary, learning from Run 1, we recommend identifying an optimal stimulation strength, while maintaining low cell concentrations, as the focus for a successful run should prioritize product quality over total cell yield.

Based on the conditions that yielded the best outcomes, we propose a final protocol for manufacturing, summarized schematically in Supplementary Figure S5. To further advance the process toward a clinical application of RevCAR Tregs in Europe, full GMP validation runs proving robustness and leading to a manufacturing license will be required. These clean-room runs have to include additional process controls and extensive quality control of the final product, including integration of microbiological safety testing for bacteria and mycoplasma, vector copy number analysis and potency, well-established for automated CAR-T production and nicely illustrated in [84].

The preserved expression of FOXP3 and Helios, along with the absence of IL-2 and IL-17A production, confirms the phenotypic stability of RevCAR Tregs even under pro-inflammatory conditions in vitro. These features are critical for preventing unwanted effector differentiation and maintaining suppressive function in vivo. Expanded RevCAR Tregs showed low to intermediate granzyme B expression that tend to increase following stimulation, consistent with previous studies [49, 50]. In murine models, granzyme B expression by Tregs has been linked to strong suppressive activity [50], graft survival [85] and control of lung inflammation [86]. Its upregulation under activation and inflammatory conditions may therefore reflect a functional shift toward more potent suppression [87]. Low to moderate levels of IFN-γ and TNF-α were also detected, although their functional implications remain unclear. IFN-γ is known to be expressed by both freshly isolated and expanded FOXP3^+^ Tregs [88], with evidence also supporting its regulatory function against donor allogenic antigens [89], and may therefore define a natural physiological subset. The expression of TNF-α by ex vivo expanded Treg products has been reported before, however, the pleiotropic nature of this cytokine suggests that its function is likely context-dependent and needs further investigation [90–92].

The observed upregulation of the early activation marker CD69 exclusively in the presence of CEA-IgG4-5B9 RevTM and CEA^+^ target cells confirm antigen-specific RevCAR Treg activation. This is critical for minimizing off-target effects and increasing in vivo Treg persistence as shown in a recent study by Uenishi et al. (2024) [93]. To further evaluate the specificity of RevCAR Treg interactions, adhesion measurements were performed using AFM-SCFS. In this assay, RevCAR Tregs exhibited stronger adhesion to CEA^+^ target cells in the presence of the specific RevTM in comparison to conditions with irrelevant RevTM. Previous studies have established that CAR T cells form non-classical immunological synapses, where mechanical forces drive CAR activation and downstream signaling [48]. In line with this, our AFM-SCFS data suggest that RevCAR Tregs similarly exploit force-based mechanisms during activation. Although the AFM-SCFS served as a proof-of-concept study conducted with one product (Run 1), our findings demonstrate its value as a highly sensitive approach for quantifying CAR Treg interactions. Taken together, the specificity observed at both functional and biophysical levels suggests the potential for RevCAR Tregs to selectively localize and become activated within the target tissues. Most importantly, our suppression assay data demonstrate that RevCAR Tregs exert effective, dose-dependent suppression of Teff proliferation and expansion, confirming their functional capacity following transduction and ex vivo propagation.

### Conclusion

In this study, we established a clinical-scale, GMP-compatible manufacturing process for universal adapter RevCAR Tregs, yielding a highly pure product at a clinically relevant dose. The process achieved highly efficient viral transduction for genetic modification, and the resulting RevCAR Tregs expressed markers indicative of a stable, functional phenotype with strong homing capacity. Importantly, we demonstrate that RevCAR Tregs are specifically activated in the presence of target antigen and RevTM and display potent, dose-dependent suppressive activity. This represents a crucial step toward the clinical translation of RevCAR Tregs.

Off-the-shelf cell therapies offer a rapid treatment option by eliminating the need for patient-specific manufacturing. Building on this advantage, the RevCAR Treg platform has the potential to provide additional flexibility and accessibility through the banking of RevTMs with different specificities. Moreover, the prospective banking of RevCAR Tregs from HLA-homozygous donors may support the development of a universal, allogeneic therapy applicable to a broader patient population. However, we acknowledge the challenge of accessing said rare starting material through donor registries. We believe an off-the-shelf approach will reduce costs, shorten manufacturing timelines and support wider clinical application of RevCAR Tregs.

## Methods

### Gene constructs and cell lines

#### Gene constructs

The novel CEA-IgG4-5B9 RevTM was generated by fusing an scFv [26, 94] with specificity for CEA via the hinge, C_H_2 and C_H_3 domain of a mutated human IgG4 antibody [26] to an scFv specific for the E5B9 peptide epitope (derived from the anti-La monoclonal antibody 5B9) of the RevCAR [95]. The resulting construct was cloned into the lentiviral vector p6NST50. In addition, the CEA-IgG4-7B6 RevTM without specificity for this RevCAR [26], was used as a control. Both RevTMs contain an Igk signal peptide (SP) for secretion into the cell culture supernatant as well as a Strep- and His-tag for protein purification and detection. The in-house generation of the research-grade RevCAR construct was outlined in detail in Feldmann et al. 2020 [23].

#### Cell lines

HT-29 cells were obtained from German Collection of Microorganisms and Cell Cultures (DSMZ), whereas 3T3 and HEK-293T cells were purchased from American Type Culture Collection (ATCC). Generation of 3T3 cells producing the CEA-IgG4-7B6 RevTM and HT-29 CEA^high^Luc^+^ cells expressing human CEA and firefly luciferase (Luc) was outlined in Soto et al. 2023 [26]. For permanent expression of the novel CEA-IgG4-5B9 RevTM, 3T3 cells were genetically modified with lentiviral particles produced in HEK-293T cells as previously described [96–98]. 3T3 and HEK-293T cells were cultured in DMEM complete media, whereas HT-29 CEA^high^Luc^+^ cells were cultured in RPMI complete medium as defined in our previous work [20]. All cells were maintained at 37 °C in a humidified atmosphere having 5% (v/v) CO_2_ and regularly tested for being mycoplasma negative.

### Manufacturing

This paper presents data in accordance with the minimum information about T regulatory cells (MITREG) guidelines outlined in our previously published work [99] to ensure standardized reporting on Treg manufacturing. A total of five independent manufacturing runs were completed and the following statistics are reported as median values with corresponding ranges.

#### Apheresis

The apheresis procedure was performed in the Department of Transfusion Medicine, Carl Gustav Carus University Hospital at TUD Dresden University of Technology, after obtaining informed consent from healthy human volunteers (ethics approval file number EK − 80022019). A continuous-flow cell separator, Spectra Optia^®^ (Terumo BCT), was used for the procedure. Fresh, non-mobilized apheresis products were collected from five volunteers aged 22 to 25 years, weighing between 65 and 92 kg and supplemented with ACD-A and optionally heparin as anticoagulants. The apheresis material was transported to the GMP facility within 20 minutes at ambient temperature and processing started within 1 hour. The total number of nucleated cells was determined using MACSQuant^®^ Analyzer 10 (Miltenyi Biotec) after staining for 10 minutes with a CD45–V500 antibody (Clone HI30, BD Biosciences) at room temperature (RT). The apheresis products had a median volume of 166 mL (range: 120–260 mL) with a CD45^+^ cell concentration of 100 × 10^6^ cells/mL (range: 68.4 × 10^6^ −148 × 10^6^ cells/mL).

#### Clinical scale CD25 immunomagnetic enrichment

Apheresis volume containing 1 × 10^10^ CD45^+^ cells (except Run 3: 0.83 × 10^10^) was processed as per manufacturer’s protocol. In brief, the cells were diluted to a final volume of 600 mL with working buffer composed of CliniMACS PBS/EDTA Buffer (Miltenyi Biotec) supplemented with 0.5% (v/v) Human Serum Albumin (HSA, Baxalta) and centrifuged at 300xg, 15 minutes to remove thrombocytes. The cells were resuspended in 30 mL of buffer and stained using MACS^®^ GMP CD25-PE-Biotin (Clone 3G10, Miltenyi Biotec) at 1:20 dilution for 10 minutes, RT with a tumbling shaker at 25 ± 5 rpm. This was followed by the incubation with CliniMACS^®^ Anti-Biotin Reagent (Miltenyi Biotec) without an intermediate wash step for 30 minutes, RT with a tumbling shaker at 25 ± 5 rpm. The cells were washed with working buffer (300xg, 15 minutes) and sorted on the CliniMACS^®^ Plus Instrument (Miltenyi Biotec) using the CliniMACS^®^ Tubing Set LS (Miltenyi Biotec) with the ‘ENRICHMENT 2.1’ program. The resulting enriched fraction containing CD25^+^ cells was resuspended in 100 mL working buffer and stored in Falcon tubes at 4 °C overnight before further processing.

#### GMP compliant fluorescence-activated cell sorting

The enriched cells were further labeled using MACS^®^ GMP clinical-grade antibodies: CD4-Vioblue (Clone M-T466, Miltenyi Biotec) and CD127-FITC (Clone MB15-18C9, Miltenyi Biotec) at 1:31 dilution and CD8-APC (Clone BW135/80, Miltenyi Biotec) at either 1:31 or 1:21 dilution for 30 minutes at 4 °C, followed by a single wash with working buffer (300xg, 15 minutes). For sorting, the cells were resuspended in 10 mL of MACSQuant^®^ Tyto^®^ Running Buffer (Miltenyi Biotec) achieving an average concentration of 7.4 × 10^6^ cells/mL (range: 6.9 × 10^6^ to 7.8 × 10^6^). The cells were filtered through a 20 µm cell-strainer and loaded into the MACSQuant^®^ Tyto^®^ high speed (HS) cartridges (Miltenyi Biotec) and sorted on the MACSQuant^®^ Tyto^®^ Cell Sorter (Miltenyi Biotec). The culture medium was prepared using TexMACS^™^ GMP Medium (Miltenyi Biotec) supplemented with 5% (v/v) ATMP-grade human serum (Center for Clinical Transfusion Medicine (ZKT), Tübingen) and 1,000 IU/mL IL-2 (Proleukin^®^, Novartis). The sorted cells with an average yield of 22.6 × 10^6^ cells (range: 16 × 10^6^ − 38.4 × 10^6^), were seeded in 70–100 mL of the culture medium in a 100 mL MACS^®^ GMP Cell Differentiation bag (Miltenyi Biotec) and stored at 37 °C overnight before initiating the expansion process.

#### Automated closed system transduction and expansion

For the transduction and expansion process, the CliniMACS Prodigy^®^ Tubing Set TS 520 (Miltenyi Biotec) was installed on the CliniMACS Prodigy^®^ System (Miltenyi Biotec) using an automated protocol modified from the T cell transduction (TCT) program. Sorted cells were transferred from the cell bag into the culture chamber, where they were washed and resuspended in 70 mL of fresh culture medium. Stimulation was initiated on Day 0 using MACS^®^ GMP T Cell TransAct^™^ Large Scale reagent (Miltenyi Biotec) at a 1:52.5 dilution (referred to as 100%). Lentiviral transduction was performed either on Day 2 or Day 5 using lentiviral particles produced as previously described [96, 97] at a multiplicity of infection (MOI) of 4, followed by centrifugation in the CentriCult^®^ unit for 120 minutes at 32 °C. Forty-eight hours post-transduction, cells were washed to remove the residual virus. On Day 8, a volume reduction was performed, followed by restimulation with MACS^®^ GMP T Cell TransAct^™^ Large Scale at the same 1:52.5 dilution (100%). Restimulations were modified for Run 1 (130%) and Run 2 (65%). Fresh culture medium was added at two intervals between Day 9 and Day 12 to reach the maximum culture volume of 250 mL. In two independent runs, a wash step was performed at either 48 hours (Run 2) or 24 hours (Run 3) before harvest to remove residual TransAct and provide a resting period. Automated harvest with a formulation solution (0.9% (w/v) Sodium Chloride, Fresenius Kabi + 1% (w/v) HSA) was conducted on Day 14 (Run 2, 4 and 5) or Day 15 (Run 1 and 3). Throughout the culture period, aliquots were collected every 2–3 days using Sampling Adapters (Miltenyi Biotec) and analyzed on MACSQuant^®^ Analyzer 10 to assess cell number, viability and transduction efficiency. The harvested RevCAR Tregs were either used to set up in vitro assays or cryopreserved using Cryostor^®^ CS10 cryopreservation media (Merck).

### Phenotyping

#### Flow cytometry

Phenotyping was performed to assess Tregs following isolation and expansion using markers for Treg identification (CD3, CD4, CD25, CD127, FOXP3) and markers to detect potential cell contaminations (CD45, CD8, CD56, CD19). Antibody details are provided in Supplementary Table 1. Cells were stained with surface antibodies for 45–60 minutes at 4 °C, followed by fixation and permeabilization using the eBioscience FOXP3/Transcription Factor Staining Buffer Set for 45 minutes at RT, according to the manufacturer’s instructions (ThermoFisher Scientific). Permeabilized cells were then stained with the intracellular antibody FOXP3 for 30–45 minutes at 4 °C. Tregs were also analyzed for their RevCAR expression. For this purpose, cells were incubated with 0.25 µg of the anti-La monoclonal antibody (5B9) [95] for 1 hour at 4 °C followed by a staining with a PE goat anti-mouse IgG (minimal cross-reactivity) antibody for 30 minutes at 4 °C. After staining, cells were resuspended in the staining buffer (PBS with 0.5% (w/v) FBS, Merck) and either analyzed immediately or stored overnight before analysis on the MACSQuant^®^ 10 Analyzer. After each staining step, cells were washed twice with the staining buffer. Phenotyping of the harvested RevCAR Treg products included Florescence Minus One (FMO) control to allow precise gating for CD127 and FOXP3. The data was analyzed using FlowJo software (BD Life Sciences).

#### Mass cytometry

To achieve a more comprehensive phenotypic characterization, RevCAR Treg products were analyzed using a 29-marker mass cytometry panel on cryopreserved, post-thaw samples. Antibody details are provided in Supplementary Table 2. Cryopreserved RevCAR Tregs were thawed into X-VIVO^®^ 15 medium (Lonza) supplemented with 10% FBS. Cells were first blocked with Human FcR blocking reagent (Miltenyi Biotec) for 10 minutes at RT. To achieve homogeneous staining, each sample (up to 3 × 10^6^ cells) was uniquely barcoded using a 6-choose-3 barcoding scheme, consisting of anti-CD45 antibodies conjugated to different cadmium (Cd) and yttrium (Yt) metal isotopes (^89^Y, ^106^Cd, ^110^Cd, ^111^Cd, ^114^Cd, ^116^Cd). The barcoded samples were pooled and processed further as a single sample for surface and intracellular staining. Following viability staining with Cell-ID cisplatin (1:1000, 5 minutes at RT) (Standard BioTools), cells were stained with surface antibodies in Maxpar Cell Staining Buffer (Standard BioTools) for 45 minutes at RT, then fixed and permeabilized using the FOXP3/Transcription Factor Staining Buffer Set for 45 minutes at RT. Intracellular staining with FOXP3 and CTLA-4 antibodies was performed for 45 minutes at RT. For cell identification, samples were labeled with DNA intercalators (^191^Ir and ^193^Ir, Standard BioTools), resuspended in Maxpar Fix and Perm Buffer (Standard BioTools) with 4% methanol-free paraformaldehyde (PFA, ThermoFisher Scientific), and incubated overnight at 4 °C.The cells were cryopreserved in FBS containing 10% (v/v) DMSO and stored at −80 °C until analysis. After each staining step, cells were washed twice with Maxpar Cell Staining Buffer. Thawed samples were washed with Maxpar Water (Standard BioTools), aliquoted and stored as pellets until acquisition on the CyTOF XT mass cytometer (Standard BioTools). Data clean-up and analysis was performed using CyTOF^*®*^ software (Standard BioTools) and the cloud-based software OMIQ (Dotmatics). Debarcoding to obtain individual “.fcs” files was performed on RStudio using CATALYST package (Posit Software, PBC).

### Functional assays

#### Activation assay

To check specific activation, 1 × 10^5^ of the harvested RevCAR-Treg cells were cultured either alone or co-cultured with 2 × 10^4^ HT-29 CEA^high^Luc^+^ target cells in triplicates in the presence or absence of 5pmol RevTM. After 24 hours, the cells of the triplicates were pooled, stained with antibodies CD4-PE-Vio770 (Clone REA623, Miltenyi Biotec) and CD69-APC (Clone REA824, Miltenyi Biotec) and analyzed on MACSQuant^®^ Analyzer 10. An FMO control was used to set gates for CD69 marker. The data was analyzed using MACSQuantify^™^ Software (Miltenyi Biotec).

#### Atomic force microscopy-based single-cell force spectroscopy (AFM-SCFS)

To obtain a pure population of transduced cells for the assay, harvested RevCAR Tregs were stained with CD4-VioGreen^™^ (Clone REA623, Miltenyi Biotec), CD25-PE (Clone 4E3, Miltenyi Biotec) and CD127-APC (Clone MB15-18C9, Miltenyi Biotec). Cells were then sorted based on CD4^+^CD25^high^CD127^low^EGFP^high^ expression and the resulting EGFP^high^ RevCAR Tregs were subsequently cryopreserved.

One to two days prior to the assay, HT-29 CEA^high^Luc^+^ target cells were plated in 40 mm plastic petri dishes at the concentration of 0.1 × 10^6^ cells/mL in RPMI complete medium and cultured at 37 °C to allow adherence. Cantilevers (PNP-TR-TL-Au type B, nominal spring constant of 0.08 N/m) (Nanoworld) were plasma-cleaned (air plasma, 2 minutes) and coated overnight at 4 °C with 1 mg/ml Concanavalin A (ConA, Sigma) in PBS to facilitate cell attachment. On the day of the assay, EGFP^high^ RevCAR Tregs were thawed and pre-incubated with CEA-IgG4-5B9 RevTM or CEA-IgG4-7B6 RevTM in a 96-well U-bottom plate in RPMI complete medium for 60–90 minutes. Immediately prior to imaging, the culture medium in the petri dish containing the HT-29 CEA^high^Luc^+^ target cells was replaced with CO_2_-independent medium (ThermoFischer Scientific) + 300 IU/mL IL-2. The ConA-coated cantilevers were washed with PBS and calibrated using the thermal noise method. To begin analysis, the RevCAR Treg-RevTM mixture was transferred onto the petri dish containing the target cells and placed in a petri dish heater set to 37 °C. To pick up a RevCAR Treg pre-incubated with either of the RevTM, the cantilever tip was positioned over a single cell and gently brought into contact (2 seconds, 1 nN). After cantilever withdrawal, the cantilever-RevCAR Treg bond was allowed to stabilize for approximately 5 minutes before starting the first force-distance cycle. Then, the cantilever-RevCAR Treg pair was lowered at moderate speed (5 µm/sec) onto a HT-29 CEA^high^Luc^+^ target cell until a force setpoint of 2 nN was reached. After a preset contact time (5, 30 and 120 seconds), the cantilever-RevCAR Treg pair was pulled upwards until reaching its starting position and a force-distance curve was recorded. AFM-SCFS experiments were conducted using a Nanowizard I atomic force microscope (JPK instruments) which was mounted on an inverted light microscope (Axiovert 200, Carl Zeiss) equipped with a CCD Camera (Coolsnap CF, Diagnostic Instruments Inc.). Data was analyzed using the JPK data processing software (JPK instruments), measuring the detachment force and detachment energy in the retraction curve after correcting for baseline drift.

#### Stability assay

RevCAR Tregs (cryopreserved, post-thaw) were cultured at a density of 1 × 10^6^ cells/mL in RPMI complete medium supplemented with 300 IU/mL IL-2 and subjected to a pro-inflammatory environment under different stimulation conditions. Polyclonal stimulation was performed using Dynabeads^™^ Human T-Activator CD3/CD28 (ThermoFischer Scientific) at a 1:5 bead-to-Treg ratio in the presence or absence of a pro-inflammatory cytokine cocktail composed of IL-1β, IL-12, IL-23 and IL-6 (10 ng/mL each) along with TNF-α (20 ng/mL) (all purchased from Miltenyi Biotec). Antigen-specific activation was induced through co-culture with HT-29 CEA^high^Luc^+^ target cells and CEA-IgG4-5B9 RevTM (set-up identical to the activation assay), similarly with or without the same pro-inflammatory cytokine cocktail. After 48 hours of culture, a 4.5-hour stimulation with 25 ng/mL of phorbol 12-myristate 13-acetate (PMA, Merck) and 1 µg/mL of ionomycin (Merck) in the presence of brefeldin A (ThermoFischer Scientific) was performed, followed by an intracellular cytokine staining using the Inside Stain Kit (Miltenyi Biotec). Cells were incubated with antibodies targeting IL-2, IL-17A, IFN-γ, TNF-α or granzyme B. Intracellular staining to assess FOXP3 and Helios expression utilized FOXP3/Transcription Factor Staining Buffer Set as described above along with antibodies against FOXP3 or Helios. Antibody details are provided in Supplementary Table 3. All samples were run on BD LSR Flow Cytometer (BD Biosciences) and analyzed using FlowJo software.

#### Suppression assay

To assess the suppressive capacity of RevCAR Tregs, a polyclonal suppression assay was performed using allogenic or autologous CD4^+^CD25^-^ Teffs isolated and cryopreserved from donor buffy coats or apheresis products, obtained from the local blood collection center Deutsches Rotes Kreuz Nord-Ost. Teffs were isolated via a two-step isolation process, as described previously [39]. RevCAR Tregs (cryopreserved, post-thaw samples) and Teffs were stained with eFluor670 and eFluor450 proliferation dyes (ThermoFisher Scientific), respectively, and co-cultured in triplicates at varying ratios in the presence of Dynabeads^™^ Human T-Activator CD3/CD28 (Beads:Cells = 1:80, for allogeneic suppression assay) or T Cell TransAct^™^ (1:100, for autologous suppression assay) in RPMI complete medium for four days at 37 °C. Following incubation, cells were stained with 7-AAD (Miltenyi Biotec) and were analyzed using MACSQuant^®^ 10 Analyzer and MACSQuantify Software. Two read-outs were obtained for Teffs: (1) % proliferation, determined by the percentage of eFluor450^dim^ cells within the total eFluor450^+^ population (used to calculate % suppression at each ratio according to the formula below), and (2) n-fold expansion, based on the total eFluor450^+^ cell counts measured on Day 4 relative to Day 0.

$$\begin{aligned}\% {\rm{Suppression}}\left( {{\rm{xratio}}} \right) =& {\matrix{\% {\rm{Proliferation}}\left( {0:1{\rm{ratio}}} \right) \hfill - \% {\rm{Proliferation}}\left( {{\rm{xratio}}} \right) \hfill \cr} \over {\% {\rm{Proliferation}}\left( {0:1{\rm{ratio}}} \right)}}\times 100 \end{aligned}$$

By using GraphPad Prism 10 (GraphPad Software Inc.), a nonlinear four-parameter dose-response curve model was applied to % suppression values. The number of Tregs required for 50% suppression of Teffs was obtained from the dose-response curve and converted into a Treg:Teff_50%_ ratio based on the fixed Teff count (5 × 10^4^ cells).

### Biopsy immunofluorescence staining

Colon biopsies were obtained from an aGvHD patient with grade 3–4 disease of the colon duodenum (ethics approval file number BO–EK-11012020). Immunohistochemistry was performed on 3–5 µm formalin-fixed paraffine-embedded (FFPE) tissue sections using tyramide signal amplification-based OPAL technology (Akoya Biosciences) on the Ventana Ultra Instrument (Ventana Medical Systems) to detect CEA-expressing cells as previously described [100, 101]. Tissue sections were deparaffinized, rehydrated in Easy Prep solution (Ventana Medical Systems), and subjected to heat-mediated antigen retrieval at 95 °C for 32 minutes in Cell Conditioning Solution 1 (Ventana Medical Systems). Sections were incubated with mouse anti-human CD66e primary antibody (Clone CD30, Cell Signaling Technologies) for 32 minutes at 36 °C, followed by OmniMAP HRP anti-mouse secondary antibodies (Ventana Medical Systems) for 12 minutes at 36 °C. Opal620 fluorophore (Akoya Biosciences) was applied for 8 minutes at RT. Antibody stripping was performed by heating samples in Cell Conditioning Solution 2 (Ventana Medical Systems) at 100 °C for 24 minutes. Staining was completed with DAPI counterstaining (Merck), and sections were mounted in Fluoromount-G^®^ medium (SouthernBiotech). The images were acquired on the Vectra 3 multi-spectral imaging system (Akoya Biosciences). Spectral unmixing of images was performed with inForm^®^ software (version 2.6; Akoya Biosciences) and a representative image was prepared using Fiji software (version 2.9) [102].

### RevTM purification and biochemical characterization

4 × 10^6^ RevTM-secreting 3T3 cells were seeded into 175 cm^2^ flasks containing 50 mL of DMEM complete medium and cultured at 37 °C with 5% CO_2_. After 4 days, the supernatant was harvested and RevTMs were isolated via their Strep-tag using a Strep-Tactin^®^XT buffer set and columns (IBA-Lifesciences) as outlined in the manufacturer’s instructions. Purified RevTMs were dialyzed against PBS (1x) at 4 °C, either overnight or over the weekend, using a semi-permeable membrane (Molecular weight cut-off: 14kDa). To estimate yield and purity of isolated RevTM constructs, an SDS-PAGE was performed, followed by incubation of the gel in Quick Coomassie^®^ Stain (Serva Electrophoresis) for 1 hour on a shaker and imaging using a ChemiDoc^™^ XRS Imaging System (Bio-Rad) with Image Lab^™^ Software (Bio-Rad). In addition, immunoblotting was performed as outlined in Kegler et al. 2023 [103] and RevTMs were detected using an anti-penta-His antibody (Qiagen; 1:1,000 dilution in blocking solution, 1 hour at RT) and an alkaline phosphatase-conjugated anti-mouse IgG antibody (Dianova GmbH; 1:1,000 dilution in blocking solution, 1 hour at RT), followed by incubation with a NBT/BCIP substrate solution. Membranes were imaged using the ChemiDoc^™^ XRS Imaging System and Image Lab^™^ Software.

### Statistical analysis

Statistical analysis was performed using GraphPad Prism 10. The specific statistical tests for each analysis are indicated in figure legends where a p-value of ≤ 0.05 was considered statistically significant. As repeated measures ANOVA does not handle missing values, we applied a mixed-effects model for statistical tests where a complete data set was not available, as implemented in GraphPad Prism 10. This approach yields results comparable to repeated measures ANOVA when no data are missing.

## Electronic supplementary material

Below is the link to the electronic supplementary material.

Supplementary Material 1

## Data Availability

The datasets used and/or analyzed during the current study are available from the corresponding authors on reasonable request.

## Abbreviations

AFM: Atomic force microscopy
aGvHD: Acute graft-versus-host disease
ATMP: Advanced Therapy Medicinal Product
CAR: Chimeric antigen receptor
CEA: Carcinoembryonic antigen
CSD: Co-stimulatory domain
CM: Central memory
Con A: Concanavalin A
EM: Effector memory
FACS: Fluorescence-activated cell sorting
GI: Gastrointestinal
GMP: Good manufacturing practice
GvHD: Graft-versus-host disease
HLA: Human leukocyte antigen
HSCT: Hematopoietic stem cell transplantation
MACS: Magnetic-activated cell sorting
N: Naïve
NLM: Naïve-like memory
RevCAR: Reverse CAR
RevTM: RevCAR Target Module
SCFS: Single-cell force spectroscopy
SD: Signaling domain
scFv: Single-chain fragment variable
Teff: T effector cell
Th: T helper cell
T1D: Type 1 diabetes
Tregs: Regulatory T cells
