# Supplementary material for: Automated GMP-compatible production of universal CAR Tregs for organ-targeted tolerance induction
Source: J Transl Med. 2025 Dec 17;23:1399. doi: 10.1186/s12967-025-07431-0 (PMC12709696; doi:10.1186/s12967-025-07431-0)
Supplement: Supplementary file 1 — Supplementary Material 1 [file 12967_2025_7431_MOESM1_ESM.docx]

# Supplementary Material

Supplementary Table 1: Flow cytometry panel for phenotyping

| Marker | Fluorochrome | Clone | Company |
| --- | --- | --- | --- |
| CD127 | FITC, APC | MB15-18C9 | Miltenyi Biotec |
| CD19 | PE-Vio*^®^* 770 | REA675 | Miltenyi Biotec |
| CD25 | PE | 3G10, 4E3 | Miltenyi Biotec |
| CD3 | APC-Vio*^®^* 770 | BW264/56 | Miltenyi Biotec |
| CD4 | VioBlue*^®^* | M-T466 | Miltenyi Biotec |
| CD45 | V500 | HI30 | BD Biosciences |
| CD56 | PerCP-Cy^™^5.5 | NCAM16.2 | BD Biosciences |
| CD8 | APC | BW135/80 | Miltenyi Biotec |
| FOXP3 | PE-Cyanine7 | PCH101 | eBioscience |
| 5B9 monoclonal antibody | - | 5B9 | Bachmann et al. 2021 [91] |
| Goat anti-mouse IgG (minimal x-reactivity) | PE | Poly4053 | BioLegend |

Zombie Red™ Fixable Viability Kit (BioLegend) was used to stain and gate out dead cells.

Supplementary Table 2: Mass cytometry panel for extended phenotyping

| Marker | Metal Tag | Clone | Company |
| --- | --- | --- | --- |
| CD134 (OX40) | 150Nd | ACT35 | Standard BioTools |
| CD137 (4-1BB) | 159Tb | 4B4-1 | BioLegend |
| CD152 (CTLA-4) | 161Dy | 14D3 | Standard BioTools |
| CD161 | 156Gd | HP-3G10 | BioLegend |
| CD183 (CXCR3) | 163Dy | G025H7 | Standard BioTools |
| CD185 (CXCR5) | 153Eu | RF8B2 | Standard BioTools |
| CD19 | 142Nd | HIB19 | Standard BioTools |
| CD194 (CCR4) | 149Sm | L291H4 | Standard BioTools |
| CD195 (CCR5) | 171Yb | NP-6G4 | BioLegend |
| CD196 (CCR6) | 141Pr | G034E3 | Standard BioTools |
| CD197 (CCR7) | 167Er | G043H7 | Standard BioTools |
| CD27 | 158Gd | L128 | Standard BioTools |
| CD278 (ICOS) | 148Nd | C398.4A | Standard BioTools |
| CD28 | 160Gd | CD28.2 | Standard BioTools |
| CD3 | 115In | UCHT1 | BioLegend |
| CD31 (PECAM-1) | 145Nd | WM59 | Standard BioTools |
| CD4 | 174Yb | SK3 | Standard BioTools |
| CD44 | 170Er | BJ18 | BioLegend |
| CD45 (barcoding) | 89Y, 106Cd, 110Cd, 111Cd, 114Cd, 116Cd | HI30 | BioLegend |
| CD45RA | 155Gd | HI100 | Standard BioTools |
| CD45RO | 165Ho | UCHL1 | Standard BioTools |
| CD57 | 113In | HNK-1 | BioLegend |
| CD62L (L-selectin) | 166Er | DREG-56 | BioLegend |
| CD69 | 146Nd | FN50 | BioLegend |
| CD8a | 168Er | SK1 | Standard BioTools |
| CD95 (Fas) | 164Dy | DX2 | Standard BioTools |
| FOXP3 | 162Dy | PCH101 | Standard BioTools |
| TCRγδ | 152Sm | 11F2 | Standard BioTools |
| TIGIT | 154Sm | MBSA43 | Standard BioTools |

The antibodies purchased from BioLegend were purified unconjugated antibodies. Based on requirement they were conjugated in-house using Maxpar® X8 Multimetal Labeling Kit.

Supplementary Table 3: Flow cytometry panel for stability assay

| Marker | Fluorochrome | Clone | Company |
| --- | --- | --- | --- |
| CD127 | BV786 | HIL-7R-M21 | BD Biosciences |
| CD25 | BUV737 | 2A3 | BD Biosciences |
| CD25 | BV421 | 2A3 | BD Biosciences |
| CD3 | APC-Vio*^®^* 770 | BW264/56 | Miltenyi Biotec |
| CD4 | BUV395 | RPA-T4 | BD Biosciences |
| CD4 | VioGreen^™^ | REA623 | Miltenyi Biotec |
| CD8 | APC | BW135/80 | Miltenyi Biotec |
| FOXP3 | PE-Cyanine7 | PCH101 | eBioscience |
| Granzyme B | PE | GB11 | BD Biosciences |
| Helios | PE | 22F6 | BD Biosciences |
| IFN-γ | APC-R700 | B27 | BD Biosciences |
| IL-17A | BV421 | N49-653 | BD Biosciences |
| IL-2 | PE-Cy7 | MQ1-17H12 | BioLegend |
| TNF-α | BV711 | MAb11 | BioLegend |

Zombie Red^™^ Fixable Viability Kit (BioLegend) was used to stain and gate out dead cells.

Supplementary Table 4: Summary of manufacturing parameters and protocol adaptations across five clinical-scale runs

| **Run** | **1** | **2** | **3** | **4** | **5** |
| --- | --- | --- | --- | --- | --- |
| Apheresis Donor (Sex, Weight, Age) | M, 90 Kg, 23yrs | M, 72 Kg, 22yrs | M, 70 Kg, 23yrs | M, 92 Kg, 25yrs | M, 65 Kg, 25yrs |
| Day of Transduction  (MOI: 4) | Day 5 | Day 2 | Day 2 | Day 2 | Day 5 |
| Viability at the time of Transduction | 95.35% | 81.25% | 49.70% | 93.65% | 87.60% |
| TransAct stimulation Day 0 (1:52.5 = 100%) | 100% | 100% | 100% | 100% | 100% |
| TransAct restimulation Day 8  (1:52.5 = 100%) | 130% | 65% | 100% | 100% | 100% |
| Day 0 Treg Purity (FOXP3^+^) | 94% | 97.60% | 90.50% | 95% | 90.60% |
| Harvest Day Purity (FOXP3^+^) | 76.60% | 96% | 91.90% | 89.70% | 94% |
| Day of Harvest | Day 15 | Day 14 | Day 15 | Day 14 | Day 14 |
| Transduction efficiency (EGFP^+^, post-thaw) | 38.10% | 53.30% | 18.40% | 44.90% | 55.40% |
| Transduction efficiency (RevCAR^+^, post-thaw) | 44.00% | 64.40% | 23.50% | 60.00% | 64.00% |
| Viability on Harvest Day | 97.80% | 77.7% | 77.5% | 73.2% | 85.4% |

MOI: Multiplicity of Infection, M: Male


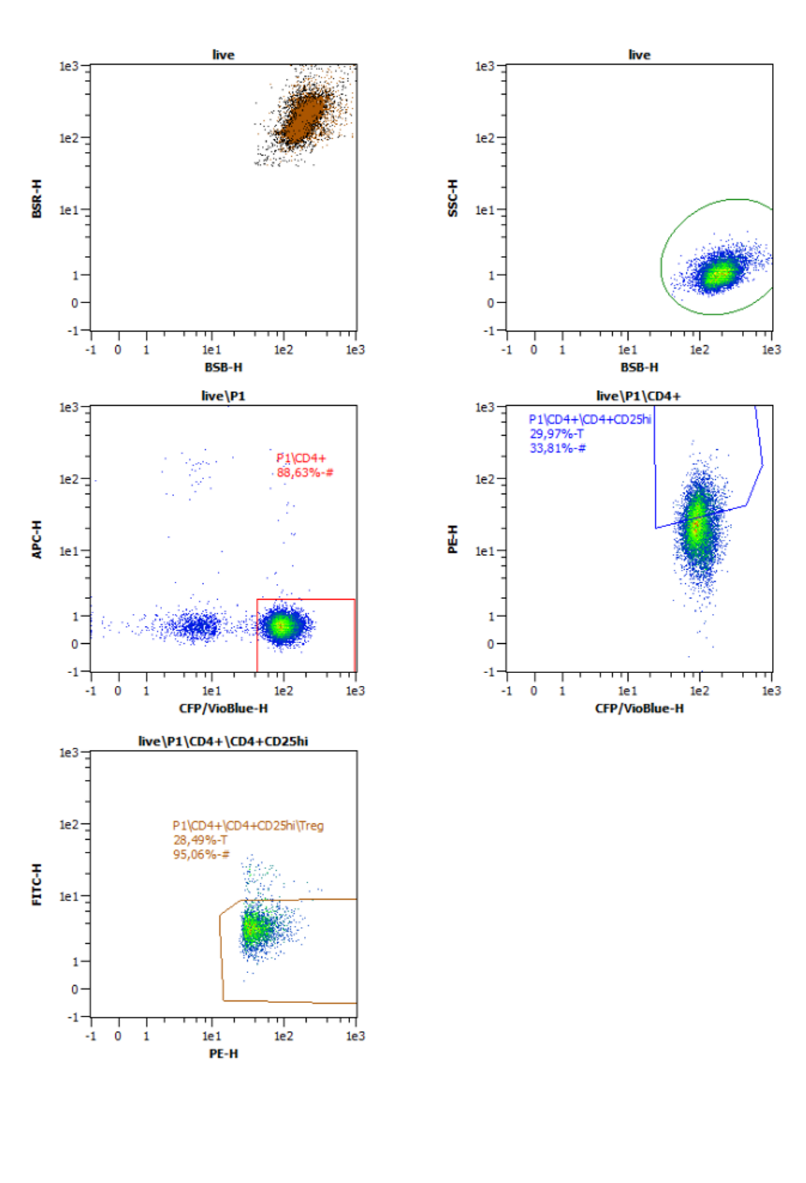


**Supplementary Figure S1:** **MACSQuant Tyto gating strategy for ‘Debulk’ sorting.** Trigger thresholds were set to exclude debris and background noise using Backscatter Red-Height (BSR-H) vs. Backscatter Blue-Height (BSB-H), followed by Side Scatter-Height (SSC-H) vs. BSB-H to gate on lymphocytes. CD4^+^ CD8^-^ T cells were gated on VioBlue-H (CD4) vs. APC-H (CD8). Within the CD4^+^ population, Tregs were identified based on high CD25 expression (PE-H), and finally gated as CD127^low^ CD25^high^ using PE-H vs. FITC-H as the final sorting gate.


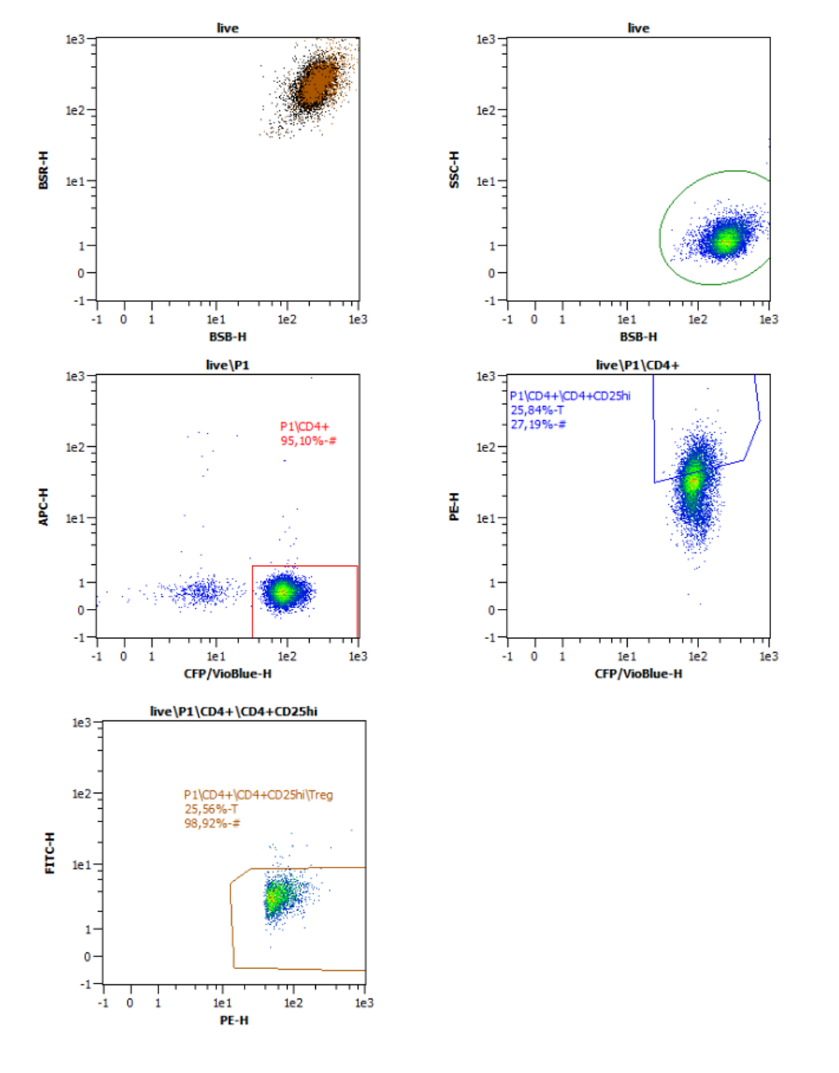


**Supplementary Figure S2:** **MACSQuant Tyto gating strategy for ‘Purity’ sorting.** Trigger thresholds were set to exclude debris and background noise using Backscatter Red-Height (BSR-H) vs. Backscatter Blue-Height (BSB-H), followed by Side Scatter-Height (SSC-H) vs. BSB-H to gate on lymphocytes. CD4^+^ CD8^-^ T cells were gated on VioBlue-H (CD4) vs. APC-H (CD8). Within the CD4^+^ population, the CD25 gate was set slightly stricter to gate for highly pure Tregs (PE-H), and finally gated as CD127^low^ CD25^high^ using PE-H vs. FITC-H as the final sorting gate.


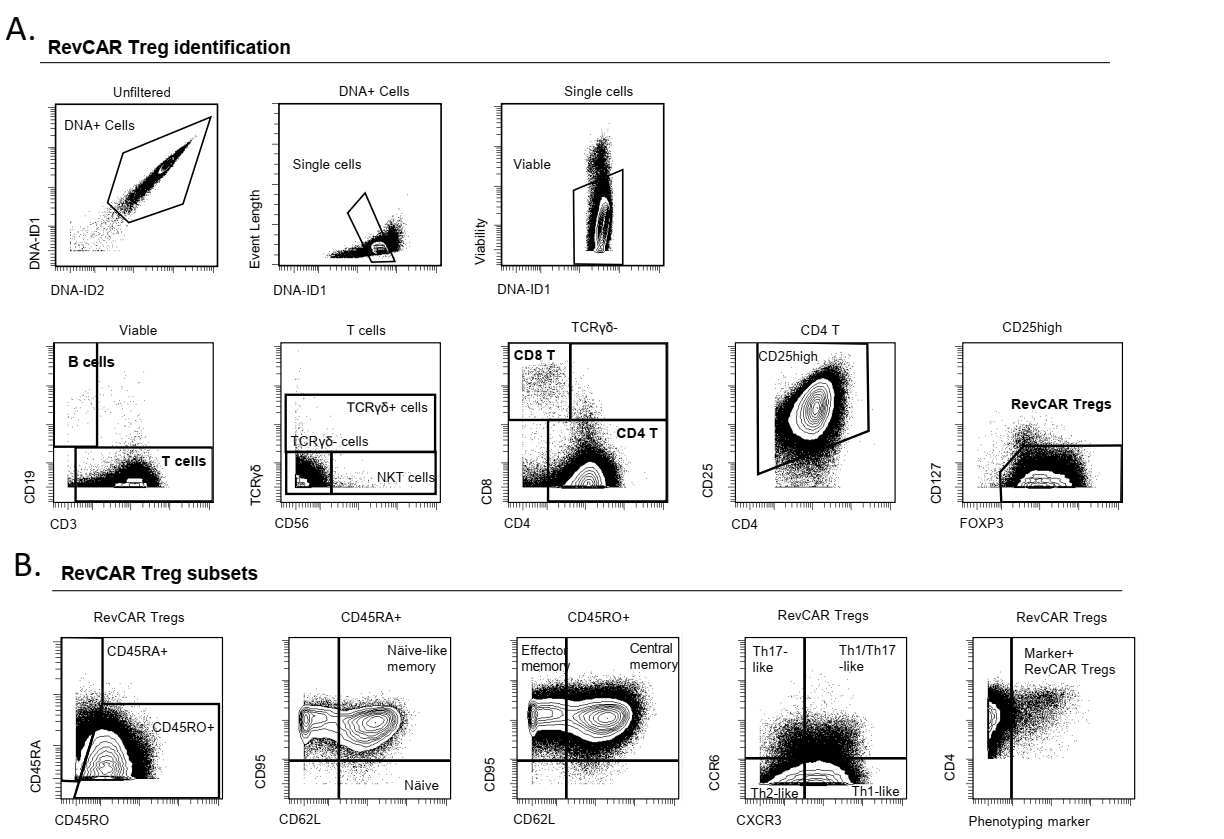


**Supplementary Figure S3:** **Mass cytometry gating strategy for comprehensive phenotyping.** Gating strategy used to identify (A) RevCAR Tregs and (B) immune subsets within RevCAR Tregs. Mass cytometry data was analyzed and visualized using the OMIQ software. Abbreviations: DNA-ID – DNA-Intercalator identification


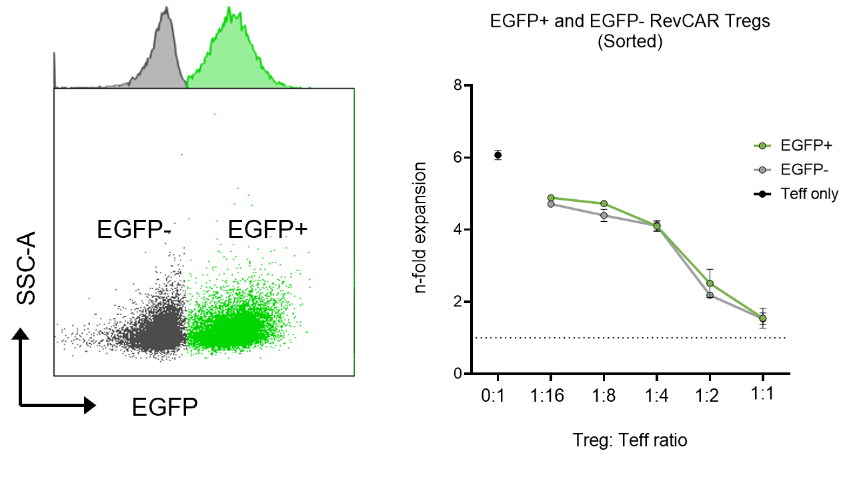


**Supplementary Figure S4: Comparison of suppressive activity between EGFP^+^ and EGFP^-^ RevCAR Tregs in a polyclonal suppression assay.** Left: Representative flow cytometry plot displaying EGFP^+^ cells (green) among single viable cells (gray). EGFP^+^ and EGFP^-^ RevCAR Treg sorted fractions displayed equal dose-dependent suppression of autologous Teff expansion upon polyclonal stimulation. Data are shown from triplicate wells and presented as mean ± SD.


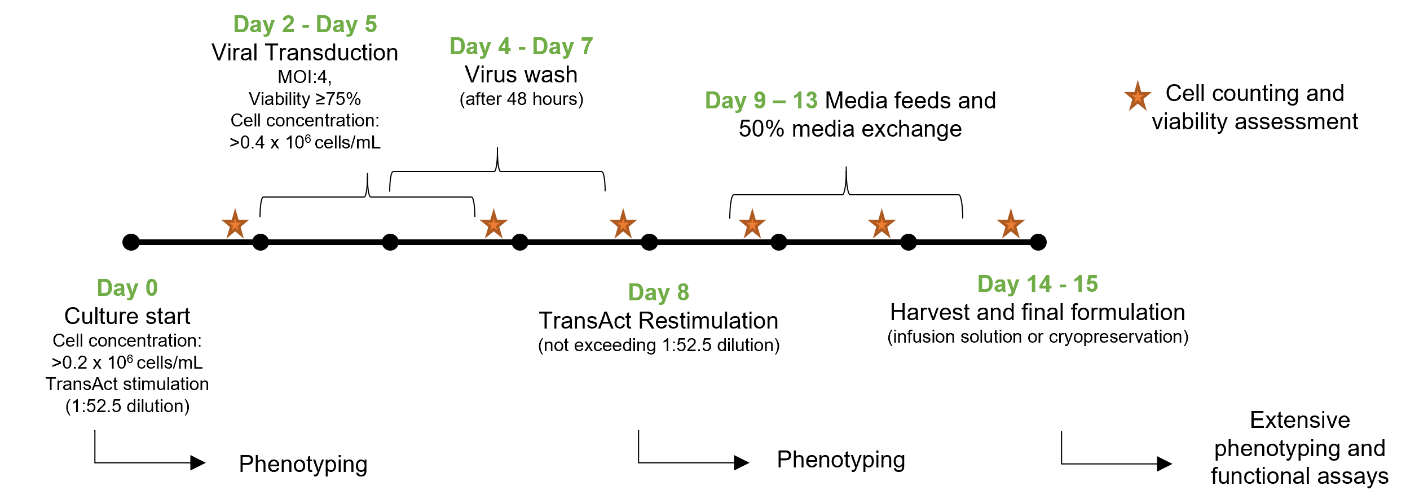


**Supplementary Figure S5: Schematic representation of the final proposed clinical-scale RevCAR Treg expansion process outlining important steps.** Culture of cells starts at Day 0, followed by transduction between Day 2 and Day 5 depending on cell viability (≥75%) followed by automated culture wash after 48 hours. At Day 8, cells are restimulated with TransAct. Media feed to be performed at two intervals between Day 9 to Day 12 with an additional 50% media exchange on Day 13. Automated harvest and final formulation of the cells takes place between Day 14 and Day 15 followed by extensive phenotyping and functional assays.
